# WNT/beta-catenin signalling interrupts a senescence-induction cascade in human mesenchymal stem cells that restricts their expansion

**DOI:** 10.1007/s00018-021-04035-x

**Published:** 2022-01-20

**Authors:** Johannes Lehmann, Roberto Narcisi, Natasja Franceschini, Danai Chatzivasileiou, Cindy G. Boer, Wendy J. L. M. Koevoet, Diana Putavet, Dubravka Drabek, Rien van Haperen, Peter L. J. de Keizer, Gerjo J. V. M. van Osch, Derk ten Berge

**Affiliations:** 1 Department of Otorhinolaryngology and Head and Neck Surgery, Erasmus MC, University Medical Center, Rotterdam, The Netherlands; 2 Department of Cell Biology, Erasmus MC, University Medical Center, Rotterdam, The Netherlands; 3 Department of Orthopaedics and Sports Medicine, Erasmus MC, University Medical Center, Rotterdam, The Netherlands; 4 Department of Internal Medicine, Erasmus MC, University Medical Center, Rotterdam, The Netherlands; 5 Center for Molecular Medicine, Section Molecular Cancer Research, Division LAB, University Medical Center Utrecht, Utrecht, The Netherlands; 6 Department of Genetics, Erasmus MC, University Medical Center, Rotterdam, The Netherlands; 7 Harbour Biomed, Rotterdam, the Netherlands; 8 Present Address: Center for Molecular Medicine, Section Molecular Cancer Research, Division LAB, University Medical Center Utrecht, Utrecht, The Netherlands

**Keywords:** Multipotent stromal cells, Cell cycle, Secondary senescence, Paracrine senescence

## Abstract

Senescence, the irreversible cell cycle arrest of damaged cells, is accompanied by a deleterious pro-inflammatory senescence-associated secretory phenotype (SASP). Senescence and the SASP are major factors in aging, cancer, and degenerative diseases, and interfere with the expansion of adult cells in vitro, yet little is known about how to counteract their induction and deleterious effects. Paracrine signals are increasingly recognized as important senescence triggers and understanding their regulation and mode of action may provide novel opportunities to reduce senescence-induced inflammation and improve cell-based therapies. Here, we show that the signalling protein WNT3A counteracts the induction of paracrine senescence in cultured human adult mesenchymal stem cells (MSCs). We find that entry into senescence in a small subpopulation of MSCs triggers a secretome that causes a feed-forward signalling cascade that with increasing speed induces healthy cells into senescence. WNT signals interrupt this cascade by repressing cytokines that mediate this induction of senescence. Inhibition of those mediators by interference with NF-κB or interleukin 6 signalling reduced paracrine senescence in absence of WNT3A and promoted the expansion of MSCs. Our work reveals how WNT signals can antagonize senescence and has relevance not only for expansion of adult cells but can also provide new insights into senescence-associated inflammatory and degenerative diseases.

**Supplementary Information:**

The online version contains supplementary material available at 10.1007/s00018-021-04035-x.

## Introduction

Following irreparable damage, healthy cells can enter a state of stable cell cycle arrest termed senescence [1]. Senescence regulates tissue growth during development and can repress expansion of transformed cells [2–4] but also limits somatic cell expansion, contributing to ageing and hampering cell-based therapies [5–8]. Several stressors can induce senescence, including shortening telomeres, DNA damage, mitochondrial deterioration, and oncogene expression [1]. Over the last decade, evidence accumulated that senescent cells affect their environment via their secretome, often termed the senescence-associated secretory phenotype (SASP) (reviewed in [9]), consisting mostly of pro-inflammatory cytokines, proteases and insulin-growth factor-binding proteins [10, 11]. A subset of SASP factors induces proliferation during wound healing and development [3, 4, 12–14]. However, in other circumstances, SASP factors mediate the spread of senescence to surrounding cells [15–21].

Recently, it has been shown that senescent mesenchymal cells can systemically induce senescence via paracrine signalling, leading to increased frailty and reduced life span [8] and reduced regenerative capacity [22], although it remains unclear how this process is regulated. Mesenchymal stem cells (MSCs, also known as multipotent stromal cells) have been intensely studied for tissue engineering due to their ability to form fat, cartilage and bone-like tissues [23], and for the anti-inflammatory, repair-inducing properties of their secretome [24–27]. However, MSCs rapidly undergo senescence in vitro, not only limiting the numbers that can be obtained for clinical applications [5, 28–32], but creating a source of SASP factors that can drive frailty and neoplastic progression [8, 33], obliterate the anti-inflammatory capacities of MSCs [34] and aggravate inflammation-associated diseases, such as atherosclerosis and osteoarthritis [35, 36]. Understanding the mechanisms that induce senescence in MSCs and cause the production of SASP factors will not only facilitate MSC-based clinical applications but may provide insight into the role of paracrine senescence in tissue homeostasis, regeneration, cancer and degenerative diseases.

We previously showed that WNT signals support the expansion and developmental potential of embryonic chondrogenic progenitors [37, 38] and of MSCs during prolonged culture [32, 39]. The WNT/β-catenin signalling pathway supports the self-renewal of many types of stem cells, including embryonic stem cells [40], intestinal stem cells [41], and hair follicle and epidermal stem cells [42], by inhibiting their differentiation and promoting their proliferation (reviewed in [43]). In contrast to these earlier examples, we demonstrate here that WNT signals support MSC proliferation and developmental potential not by regulating proliferation and differentiation but by protecting the cells from the deleterious effects of senescence.

## Results

### WNT3A counteracts entry of MSCs into senescence

**Fig. 1:**
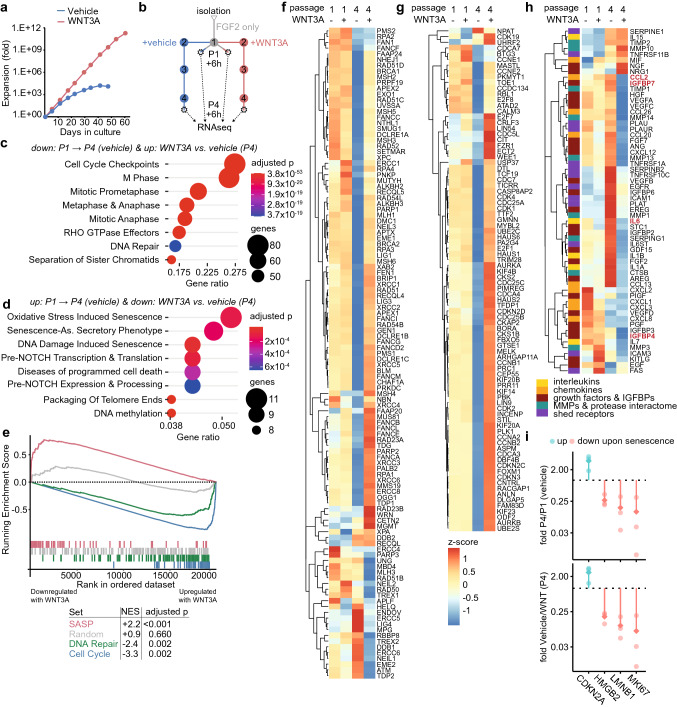
WNT3A maintains cell cycle drivers and DNA repair while repressing SASP factors. **a** Expansion of MSCs in response to WNT3A. **b** Scheme of the RNA sequencing approach. *n* = *3 donors (see Supplementary file 1 for a table of expressed genes with their fold change and multiple-testing adjusted (Benjamini–Hochberg/FDR) p-values indicated)*. **c, d** Top 8 pathways ranked according to the ratio of genes enriched to total pathway genes identified by Reactome Pathway Analysis as significantly altered (multiple-testing adjusted (Benjamini–Hochberg/FDR) *p *values < 0.001) among genes **c** down-regulated from passage one to passage four and up-regulated in MSCs expanded four passages with WNT3A compared to MSCs expanded in vehicle or **d** vice versa (s*ee Supplementary file 2 for all pathways, p values and enriched genes).* **e** Gene set enrichment analysis for sets of SASP genes [11] cell cycle associated genes [44], DNA repair associated genes [45] and size-matched set of random genes with normalized enrichment score (NES) and multiple-testing adjusted (Holms–Bonferroni) p values given. **f–h** Heatmaps showing the expression levels (z-scored) at passage one and four in MSCs expanded with WNT3A or vehicle, respectively, for the **f** the DNA repair set, **g** the cell cycle gene set and **f** the SASP gene set (*identical scale for f–h, SASP genes of interest are highlighted in red, genes are hierarchically clustered according to complete linkage*). **i** Expression of genes up- and down-regulated upon senescence in MSCs expanded with vehicle at passage four as a fold of their expression at passage one in vehicle (top) or at passage four in WNT3A (bottom). *n* = *3 donors*

The proliferation of human bone marrow-derived MSCs rapidly declines over time, and this can be counteracted by supplementation with WNT3A (Fig. 1a, Supplementary Fig. 1a) confirming our previous findings [32]. To understand how WNT signalling prevented the decline of MSC proliferation, we performed whole transcriptome analysis of early passage (P1) MSCs and of MSCs maintained until passage four (P4) with WNT3A or vehicle control (Fig. 1b). Pathway analysis identified pathways associated with DNA repair and cell cycle regulation as down-regulated with passage in vehicle and up-regulated by WNT3A (Fig. 1c), whereas pathways associated with senescence were up-regulated with passage in vehicle and down-regulated by WNT3A (Fig. 1d). To quantify these changes, we constructed gene sets for cell cycle associated genes (set based on [44]), for DNA repair regulating genes (set based on [45]) and for SASP genes (based on [10]). Gene set enrichment analysis then revealed that the DNA repair and cell cycle gene sets were significantly enriched among genes up-regulated with WNT3A, whereas the SASP gene set was significantly enriched among genes down-regulated with WNT3A (Fig. 1e). Inspection of individual gene expression levels showed that WNT3A maintained the expression levels of DNA repair and cell cycle gene sets over time in culture while they mostly declined in the absence of WNT3A (Fig. 1f, g). The majority of SASP factors (37/60), on the other hand, increased over time in the absence of WNT3A but was repressed in its presence (Fig. 1h). Impaired cell cycle progression, decline in DNA repair gene expression and the SASP are key aspects of senescence [46, 47]. Accordingly, the expression of positive and negative senescence markers (including panels from [48, 49]) was consistent with MSCs entering senescence over time, while this was counteracted by the presence of WNT3A (Fig. 1i, Supplementary Fig. 1b, c). Of note, expression of WNT family ligands and WNT/beta-catenin signalling target genes did not decrease over time in vehicle cultured MSCs (Supplementary Fig. 1d-f). This suggests that the increase in senescence is not caused by a decline in endogenous WNT ligands and is consistent with our previous findings that inhibition of endogenous WNT ligands does not affect MSC proliferation [32]. Together, these observations suggest that WNT3A counteracts the entry of MSCs into senescence.

**Fig. 2:**
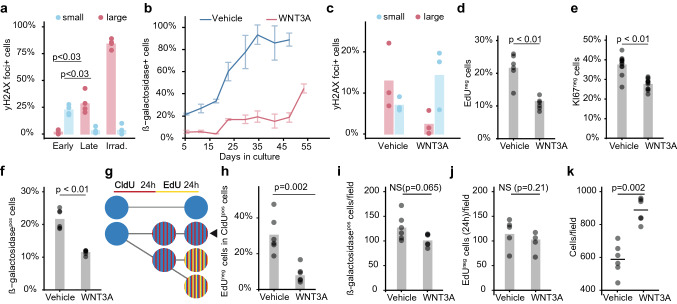
WNT3A reduces the frequency of entry into senescence. **a** Percentage of MSCs with small or large γH2AX foci after expansion for two to three passages (early) or seven to nine passages (late) or after irradiation. *n* = *4 donors, Mann–Whitney–Wilcoxon test*. See supplemental Fig. 2a for micrographs of small and large foci. **b** Percentage of cells positive for β-galactosidase in MSC populations expanded with WNT3A or vehicle over ten passages. *Error bars* = *SD, n* = *3 wells*. *Cells split from one untreated culture at day 0.* **c** Percentage of MSCs with small (< < 2 µm) or large (> 2 µm) γH2AX foci after expansion for two passages in vehicle or WNT3A. *n* = *3 wells.* Percentage of MSCs that stain **d** negative for EdU (24-h pulse) (*n* = *6 wells*), **e** negative for Ki67 (*n* = *12 wells*) or **f** positive for β-galactosidase (*n* = *6 wells*) after culture with WNT3A or vehicle for 6 days. **g** Experimental scheme for (h). **h** Percentage of cells negative for EdU (after 24-h labelling with EdU) within the population of cells positive for CldU (after 24-h labelling with CldU the day before) for MSCs cultured three passages with WNT3A or vehicle, respectively. *n* = *6 wells, Mann–Whitney–Wilcoxon test.* **I–k** Absolute number of **i** β-galactosidase-positive, **j** EdU-negative (24-h pulse) and **k** total cells after 6 days treatment with WNT3A or vehicle. *n* = *6 wells, Mann–Whitney–Wilcoxon test*

To follow up on the findings regarding DNA damage repair in our sequencing data, we stained for phosphorylated Histone 2A Family member X (γH2AX), which is elevated after ATM activation and required for double-strand break repair [50]. Late passage MSCs showed significant accumulation of cells displaying large γH2AX foci, associated with non-resolved DNA damage and the SASP [6, 51], and loss of cells displaying small foci, linked to DNA damage repair after proliferation-associated replication fork collapse (Fig. 2a, Supplementary Fig. 2a). This suggests that non-cycling cells accumulate during culture. Furthermore, we observed a rapid accumulation of cells expressing senescence-associated lysosomal β-galactosidase, an endogenous marker of senescence [52, 53] (Fig. 2b and Supplementary Fig. 2b,c). The percentage of β-galactosidase-positive cells correlated strongly with the simultaneous decline in proliferation rate over time (Supplementary Fig. 2d). WNT3A counteracted both the elevation in γH2AX-positive cells in late passage MSCs (Fig. 2c) and the accumulation of β-galactosidase-positive cells during culture (Fig. 2b and Supplementary Fig. 2b, c). Consistent with this, expression of cell cycle arrest marker CDKN1A (encoding the CDK inhibitor p21) was reduced in cells expanded with WNT3A (Supplementary Fig. 2e). Together, these data indicate that WNT3A counteracts cell cycle exit and entry into senescence of expanding MSCs.

To confirm these findings, we analysed the cell cycle dynamics of MSCs using thymidine analogue 5-Ethynyl-2'-deoxyuridine (EdU), which cells incorporate during DNA synthesis. First, we observed that early passage, WNT3A treated MSC populations undergo approximately 0.92 doublings/day (Supplementary Fig. 2f), which given losses from plating the cells suggests that the average cell cycle time is less than 24 h. This was corroborated by EdU labelling as the percentage of EdU-labelled cells rapidly increased during the first 24 h of labelling but then levelled off (Supplementary Fig. 2 g), suggesting that cycling cells had incorporated EdU within 24 h. WNT3A drastically lowered the accumulation of EdU-negative, non-cycling cells over a six-day culture period (Fig. 2d). In line with this result, WNT3A also counteracted the accumulation of cells lacking the proliferation marker Ki67 [54] (Fig. 2e). The percentage of non-cycling cells closely reflected the number of β-galactosidase-positive cells (Fig. 2d–f), suggesting they were senescent rather than quiescent cells, which remain β-galactosidase-negative [55]. The induction of senescence rather than quiescence was further supported by the upregulation of senescence-associated but not quiescence-associated genes (set based on [56, 57]) (Supplementary Fig. 2 h). By labelling cycling cells for 24 h with 5-Chloro-2-deoxyuridine (CldU), another thymidine analogue, followed by a 24-h labelling with EdU, we identified how many of those cycling cells continued cycling for a second day (as illustrated in Fig. 2g). We found that 31% of MSCs expanded for three passages exited the cell cycle within these two days, while in the presence of WNT3A only 9% of MSCs did so (Fig. 2h). As a result, the absolute number of senescent cells after six days was lower in the presence of WNT3A (Fig. 2i, j), while the total cell number was significantly increased (Fig. 2k). Altogether, these data indicate that WNT3A significantly reduces the frequency at which cells exit the cell cycle and enter into senescence, thereby maintaining expansion of the population.

### WNT/β-catenin signalling represses the senescence-inducing secretome of MSCs

**Fig. 3:**
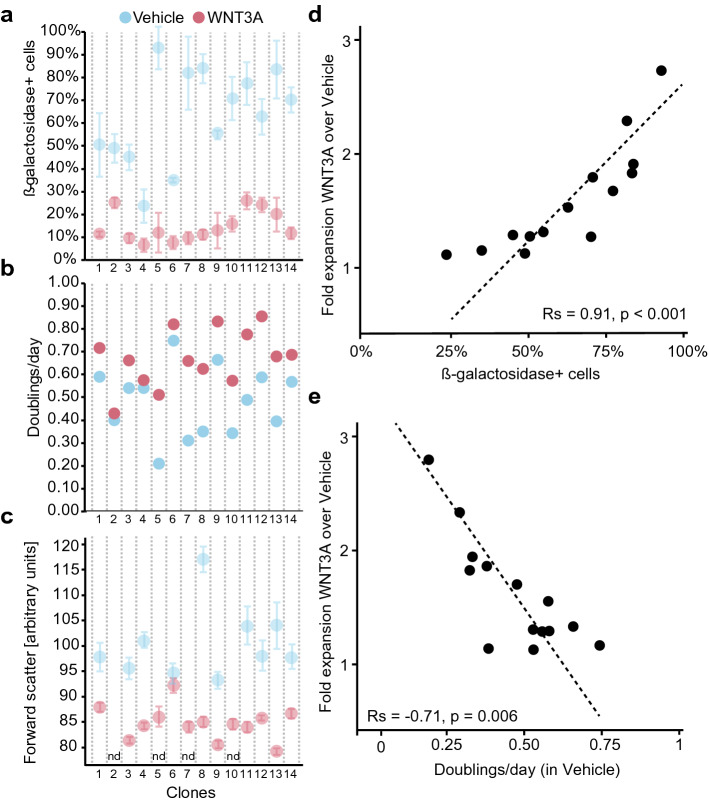
WNT represses senescence in a cell non-autonomous manner. **a–c** MSC clones obtained from a single biopsy cultured in vehicle (blue) or WNT3A (red) for one passage and analysed for **a** percentage of β-galactosidase-positive cells (*n* = *3 wells, error bars is SD)* **b** doubling rate (n = 1) or **c** forward scatter as a measure for cell size *(n* = *3 technical replicates, error bars is SD; nd: not determined due to insufficient cells in vehicle)*. **d, e** The expansion rate of MSC clones in WNT3A as fold of their expansion rate in vehicle, plotted against the **d** percentage of β-galactosidase-positive cells and **e** doubling rate in vehicle. *Rs* *Spearman’s rank correlation coefficient*

Cells can be induced to senesce by replication-based telomere shortening or other cell-intrinsic damage-associated aspects [58–61]. These driver events may be counteracted by WNT signals, e.g. through induction of telomerase to extend the telomeres, as observed in embryonic stem cells and umbilical cord MSCs [62, 63]. We did however not detect telomerase reverse transcriptase (TERT) expression in MSCs, by qPCR (Supplementary Fig. 3a, b) or by mRNA sequencing, regardless of the presence of WNT3A, which argues against a mechanism based on telomere shortening. To investigate whether WNT3A reduces senescence by selectively repressing the expansion of clones with a high intrinsic tendency of entering senescence, we established fourteen MSC clones from a single biopsy and analysed in each the progressive appearance of senescence. We found that WNT3A strongly suppressed the accumulation of β-galactosidase-positive cells in all clones (Fig. 3a), increased their proliferation rate (Fig. 3b), and prevented the cell size increase (Fig. 3c) associated with senescence [64–69]. Crucially, clones containing more senescent cells or proliferating slower showed a more pronounced increase in expansion after treatment with WNT3A (Fig. 3d, e), suggesting that WNT3A does not blunt the expansion of senescence-prone sub-clones. In addition, we observed that all clones kept accumulating β-galactosidase-positive cells at different rates even after 24–32 days of culture (Fig. 3a; Supplementary Fig. 3c), arguing against a cell-intrinsic model since senescence induced solely by intrinsic factors should co-occur within a narrow timeframe.

**Fig. 4:**
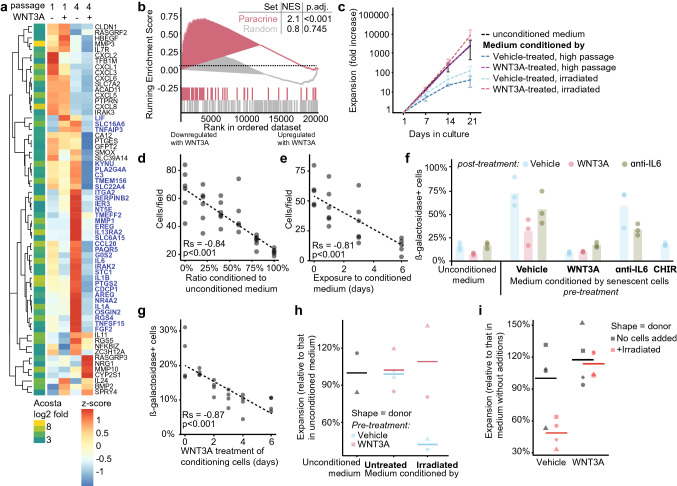
WNT/β-catenin signalling represses paracrine senescence. **a** Heatmap showing the z-scored expression levels of paracrine senescence response genes at passage one and four in MSCs expanded with WNT3A or vehicle, respectively. Genes up-regulated by time in culture and down-regulated in WNT3A-expanded MSCs are highlighted in blue. Genes are hierarchically clustered according to complete linkage. The gene set is based on genes differentially expressed in fibroblasts when co-cultured with senescent or control cells according to [18], a cut-off was applied where only genes were included that were significantly (*p* < 0.0001) up-regulated for more than ten-fold. **b** Gene set enrichment analysis for the paracrine senescence response genes and a set of random genes with normalized enrichment score (NES) and multiple-testing adjusted (Holms–Bonferroni) *p *values given. **c** Expansion of passage one MSCs in medium conditioned by MSCs rendered senescent by long-term culture or by irradiation and treated with vehicle or WNT3A or unconditioned medium not exposed to cells but otherwise processed in the same manner as conditioned medium. *Error bars* = *SD, n* = *3 donors.* **d**, **e** Cell counts for passage one MSCs after culture for **d** 6 days with the indicated ratio of conditioned medium of senescent MSCs and unconditioned medium or for **e** six, three, one or zero days in conditioned medium of senescent MSCs with the remaining time to 6 days in unconditioned medium. *n* = *6 technical replicates.* **f** Percentage of β-galactosidase-positive cells in early passage MSCs cultured for 6 days in unconditioned medium or medium conditioned by senescent MSCs that were treated with WNT3A, CHIR or an IL6-neutralizing antibody, or their pooled vehicles, respectively. In addition, WNT3A, the IL6-neutralizing antibody or their pooled vehicles were added together with the conditioned media to the recipient MSCs as indicated. *n* = *3 donors.* **g** Percentage of β-galactosidase-positive cells in early passage MSCs cultured for 6 days in medium conditioned by senescent MSCs that were treated with WNT3A for the indicated time periods. *n* = *4 donors.* **h** Expansion of low-senescence MSCs in unconditioned medium or medium conditioned by untreated or irradiated MSCs from the same population. Colours indicate whether the conditioning cells had been treated with WNT3A or vehicle. *n* = *2 donors*. **i** Expansion of low-senescence MSCs with or without addition of donor-matched irradiated MSCs in vehicle or WNT3A. Irradiated MSCs were accordingly pre-treated with either WNT3A or vehicles before addition. *n* = *4 donors*

Senescence can also be induced through extracellular signals from the SASP of neighbouring, senescent cells [18]. To explore how WNT3A affects cell-extrinsic induction of senescence, we created a paracrine senescence gene set based on publicly available microarray data of normal fibroblasts co-cultured with fibroblasts induced into senescence by the oncogene H-RAS^G12V^ [18]. Interestingly, expression of the majority of genes (32/62) in this paracrine senescence gene set increased with time in culture but was strongly reduced by the presence of WNT3A (Fig. 4a, b). This suggests that WNT3A countered paracrine senescence through reducing signalling by external factors.

To test whether secreted factors induced senescence, we exposed expanding MSCs to conditioned media from high passage senescent MSCs. As expected, this suppressed expansion in a manner dependent on both dose and duration of exposure (Fig. 4c-e). Moreover, exposure to conditioned medium increased the number of β-galactosidase-positive cells (Fig. 4f). We reasoned there to be two possibilities through which WNT3A could interfere with paracrine senescence: (a) by modulating the secretome of the senescent cells or (b) by altering the response of cells to the secretome. To distinguish between these possibilities, we first treated high passage senescent cells with WNT3A and then used those cells to prepare conditioned medium (*pre-treated* conditioned medium, which therefore did not contain added WNT3A). In addition, we prepared conditioned medium from untreated MSCs to which we added WNT3A afterwards (*post-treated* conditioned medium). *Post-treated* conditioned medium reduced, but failed to eliminate, the accumulation of β-galactosidase-positive cells (Fig. 4f). This suggests that the response to senescence-inducing factors is at least partly independent from WNT3A. Importantly, *pre-treated* conditioned medium did not suppress expansion and did not promote the accumulation of β-galactosidase-positive cells (Fig. 4c, f). These observations suggest that WNT3A represses the production of senescence-inducing factors by senescent MSCs.

We next observed that the pre-treated medium was more effective in suppressing the accumulation of β-galactosidase-positive cells if the WNT3A pre-treatment lasted longer (Fig. 4g). It could therefore simply be that WNT3A altered the secretome composition by eliminating the senescent cells from the conditioning population. To investigate this, we created a fully senescent population by exposing MSCs to ionizing radiation (Supplementary Fig. 4a). WNT3A treatment affected neither the fraction of senescent cells in the irradiated population nor its total cell number (Supplementary Fig. 4a, b). However, WNT3A pre-treatment altered the secretome of the irradiated population in such a way that it was no longer able to induce senescence (Supplementary Fig. 4c). These data argue that WNT3A suppressed the paracrine senescence-inducing secretome of senescent MSCs. If WNT3A enhanced the expansion of MSCs because it represses the paracrine signalling of senescent cells, then WNT3A should not affect expansion in absence of senescent cells. Addressing this posed a challenge because we observed some level of senescence and responsiveness to WNT3A in all adult MSCs that we tested (40 donors, data not shown). To overcome this problem, we used MSCs obtained from paediatric donors and from adult adipose tissue, both of which display little senescence at early passages as shown by us and others ([30, 70–72] and Supplementary Fig. 4d). Conditioned medium from these low-senescence MSCs had no effect on expansion of recipient MSCs (Fig. 4h). In contrast, medium conditioned by these MSCs after they were irradiated to induce senescence strongly reduced expansion of the recipient cells (Fig. 4h). In line with the earlier observations, this inhibitory effect was averted when the senescent donor cells were pre-treated with WNT3A prior to media collection (Fig. 4h). Furthermore, direct addition of irradiated cells to low-senescence cultures lowered the expansion rate of the normal cells, but this too could be rescued by treatment with WNT3A (Fig. 4i).

Finally, to determine whether the effect of WNT3A on paracrine senescence was mediated by the WNT/β-catenin pathway, we used the GSK3 inhibitor CHIR99021 (CHIR), which activates the WNT/β-catenin pathway by preventing GSK3-mediated β-catenin degradation. Similar to WNT3A, conditioned medium from senescent MSCs treated with CHIR did not induce senescence in recipient cells (Fig. 4f and Supplementary Fig. 4e). Together, these findings demonstrate that paracrine factors secreted by senescent MSCs induce entry of non-senescent MSCs into senescence, and that WNT/β-catenin signalling interferes with the production of these factors.

### WNT/β-catenin signalling represses inducers of paracrine senescence in the Senescence-associated Secretome

**Fig. 5:**
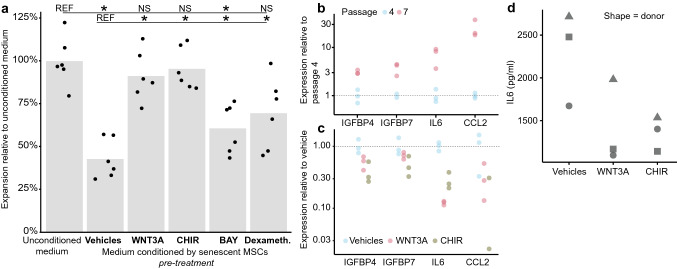
WNT/β-catenin pathway activation represses factors mediating paracrine senescence. **a** Expansion of early passage MSCs cultured in medium conditioned by senescent MSCs that were treated with WNT3A, CHIR, BAY11-7082 (BAY), dexamethasone or their pooled vehicles as a fold of expansion in control medium. *n* = *6 donors*. Wilcoxon–Mann–Whitney test against (top line) control medium or (bottom line) conditioned medium from vehicle-treated senescent MSCs. * p =  < *0.05; NS* = *p* > *0.05; p values vs. vehicle/control: Control (0.002/NA), BAY (0.031/0.031), dexamethasone (0.031/0.063), WNT3A (0.031/0.219), CHIR (0.031/0.688), vehicle (NA/0.002).* **b** Expression of selected SASP genes determined by QPCR for MSCs at passage four and passage seven. **c** Expression of selected SASP genes determined by QPCR for MSCs cultured for 24 h with vehicles, WNT3A or CHIR. *n* = *3 donors.* Expression of WNT/β-catenin signalling target genes to indicate magnitude of WNT/β-catenin pathway activation for these samples in Supplementary Fig. 5 **d** IL6 protein concentration in medium conditioned for 24 h by senescent MSCs after 24-h treatment with WNT3A, CHIR or their pooled vehicles. *n* = *3 donors*

Next, we explored whether SASP factors could be responsible for the paracrine induction of senescence in MSCs. Corticosteroids as well as inhibitors of NF-κB signalling repress a broad spectrum of SASP genes [73–75]. We found that pre-treatment of senescent cells with the corticosteroid dexamethasone as well as with the NF-κB inhibitor BAY11-7082 prior to conditioning reduced paracrine senescence in recipient MSCs, although less pronounced than pre-treatment with WNT3A or CHIR (Fig. 5a). Senescent MSCs expressed several SASP factors associated with paracrine senescence at a higher level, including cytokine interleukin 6 (IL6) and chemokine (C–C motif) ligand 2 (CCL2) as well as Insulin-like growth factor-binding protein (IGFBP) family members IGFBP4 and IGFBP7 [15, 76, 77] (Fig. 5b). Treatment of senescent MSCs with WNT3A or CHIR repressed this subset of SASP factors (Fig. 5c, Supplementary Fig. 5). Repression of SASP factors may therefore be a mechanism by which WNT3A prevents the paracrine induction of senescence.

Of these SASP factors, IL6 is thought to play a key role in maintaining SASP and paracrine senescence [75, 77–79]. WNT3A-expanded MSCs showed lower expression of IL6 compared to vehicle expanded MSCs (Fig. 1h, Supplementary Fig. 3b). In addition, WNT3A and CHIR each repressed IL6 expression within 24 h both on RNA (Fig. 5c) and protein level (Fig. 5d). We therefore investigated if interference with IL6 using an IL6-neutralizing antibody (αIL6) would reduce paracrine senescence. Indeed, addition of αIL6 to conditioned medium from senescent MSCs reduced the induction of senescence in low passage MSCs (Fig. 4f). When both the conditioning senescent cells and the recipient cells were treated with αIL6, paracrine senescence was significantly reduced—albeit not completely abolished as when both donor and recipient were treated with WNT3A (Fig. 4f). The observation that WNT3A treatment reduced senescence in cells exposed to the secretome of senescent cells (Fig. 4f) might be due to WNT3A repressing secondary paracrine senescence, whereby factors inducing paracrine senescence also induce their own expression in recipient cells (see IL6 in gene set upregulated *in response* to SASP exposure in Fig. 4a), leading to an exponential spread of senescence. Altogether these data indicate that WNT3A represses SASP factors, including IL6, which mediate the paracrine induction of senescence.

## Discussion

**Fig. 6:**
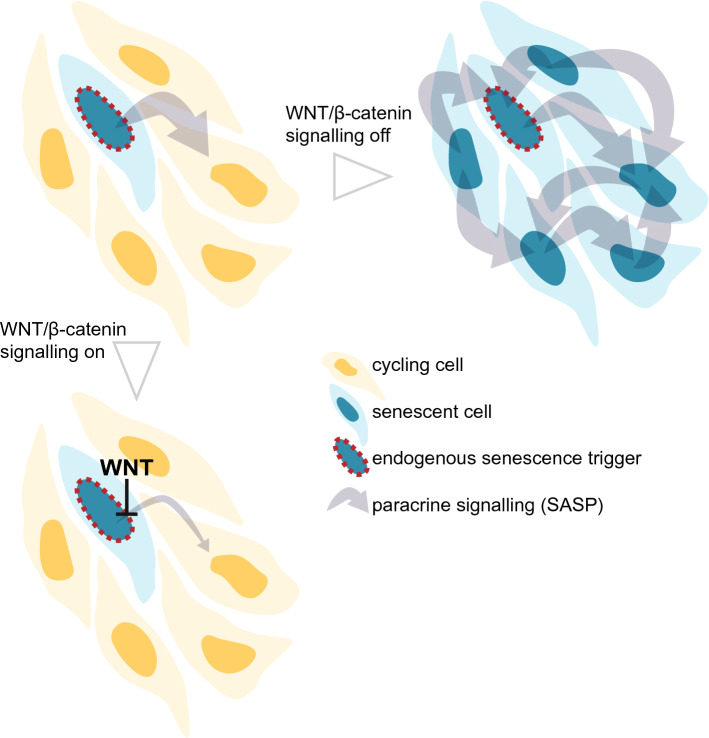
Model of the Abrogation of Paracrine Senescence by WNT/b-catenin Signalling. A cell undergoes senescence (blue) due to an endogenous trigger (red), e.g. DNA damage. Upon senescence, the cell develops the senescence-associated secretory phenotype (SASP), including factors mediating paracrine senescence, which induce senescence in surrounding cycling cells (yellow). The paracrine spread of senescence eventually renders the entire population senescent. However, activation of WNT/b-catenin signalling represses expression of the paracrine senescence mediating factors, thereby limiting the spread of senescence

The expansion and differentiation potential of MSCs decays rapidly in culture, representing a major hurdle in both research and clinical application. Here we showed that senescence spreads in MSC cultures via paracrine signalling by senescent cells displaying the SASP, leading to a self-amplifying loss of cycling cells (Fig. 6). We further demonstrated that WNT/β-catenin signals repress SASP factors and prevent paracrine senescence induction, elucidating a hitherto unknown antagonism of senescence by the WNT pathway.

MSCs are explored for their promise for tissue engineering and the potential use of their secretome to treat inflammatory and auto-immune disorders, with 50.000 patients enrolled and 10.000 treated in clinical trials employing MSCs from 2011 to 2018 [80]. However, their limited expansion potential and rapid conversion into senescence in vitro poses major hurdles for their scale-up and successful application. The pro-inflammatory secretome of senescent MSCs interferes with applications in anti-inflammatory and autoimmune disorders [34]. Moreover, the transplantation of even a small number causes adverse systemic effects and a severe reduction in health [8]. By suppressing paracrine senescence, WNT3A might not only improve clinical applicability of MSCs by facilitating their expansion but also by lowering the proportion of senescent cells and repressing the pro-inflammatory SASP. We have previously shown that WNT/β-catenin signalling agonists in combination with FGF2 maintain the differentiation potential of MSCs [32, 39]. In light of our current findings, it is plausible that senescence drives the loss in differentiation potential over time. Indeed, differentiation is tightly linked to the cell cycle [81] and SASP growth factors and interleukins have been suggested to rewire the signalling cascades guiding differentiation [13, 82, 83]. Senescence affects other adult cells in culture including hepatocytes [84], endothelial cells [85] and T cells (reviewed in [86]). Counteracting senescence-inducing factors may thus be a general strategy to support cell expansion. Combinations of WNT3A with additional SASP-inhibiting factors can be explored to maximize the expansion of MSCs and other adult cells in culture. FGF2 has been reported to delay senescence in MSCs populations [29, 87, 88] and might enhance the suppression of paracrine senescence by WNT3A (in this study all cultures were performed in the presence of exogenous FGF2). Furthermore, since WNT signals support self-renewal of many types of stem cells, counteracting senescence may have wider implications in stem cell biology.

Several studies show opposite effects of paracrine senescence and WNT signalling in neoplastic progression. While paracrine senescence limits neoplastic progression in sessile serrated adenoma and nevi [17, 18], WNT activation is associated with dysplasia in sessile serrated adenoma [89, 90] and delays senescence and undermines tumour suppression in nevi [91, 92]. In a murine colorectal cancer model, suppression of WNT signals induced a pro-inflammatory phenotype in cancer-associated fibroblasts and reduced tumour growth in a non-cell autonomous manner [93]. WNT signalling may therefore impact neoplastic progression by modulating the secretome of senescent cells, and the balance of paracrine senescence and WNT signalling could provide important diagnostic information, or even represent a treatment target.

Understanding the downstream mechanisms by which WNT signals repress SASP will be vital in understanding its role in stem cells and maximizing the potential of MSC-based therapies. Multiple studies suggest that transcription factor NF-κB regulates the majority of the pro-inflammatory SASP genes [18, 79, 94, 95]. Our findings that NF-κB signalling modulators suppressed paracrine senescence in MSCs suggest that WNT may repress NF-κB targets within the SASP set. Consistent with this, β-catenin modulates NF-κB signalling in a range of human cell lines by preventing NF-κB recruitment to the chromatin [96]. Upon induction of senescence in a cancer line of mesenchymal origin (osteosarcoma, U2-OS), phosphorylation of NF-κB subunit p65 by GSK3β paves the way to a persistent SASP because it represses transcription of IκBα, which otherwise acts as the negative feedback loop restricting NF-κB activity [97]*.* WNT3A and CHIR activate β-catenin signalling by inhibiting GSK3 activity [98] and WNT2 is repressed upon entry into oncogene-induced senescence, leading to increased GSK3β activity [99]. Although there is uncertainty on how GSK3β substrates other than β-catenin are affected by WNT ligands [98], this highlights the possibility that the repression of SASP genes we observe in response to WNT/β-catenin agonists is mediated by reduced GSK3β–NF-κB interaction. Supporting this, GSK3β inhibitor lithium chloride rescues IκBα expression ([97]) and we have previously shown that lithium chloride activates WNT/β-catenin signalling in MSCs and maintains their expansion [39]*.* Further analysis of the interaction between the WNT and NF-κB pathways may lead to novel options for repressing senescence and promoting expansion of adult cells.

To optimize MSC expansion, it is important to identify which SASP factors are the dominant factors mediating the population-wide paracrine spread of senescence. While we identify IL6 as a WNT-responsive contributor to paracrine senescence, several other secreted mediators for paracrine senescence in MSCs have been described: IGFBP4 and IGFBP7 in adult bone marrow MSCs, CCL2 in umbilical cord MSCs, and Pro-platelet basic protein (PPBP, also known as Neutrophil-Activating Peptide 2) and the hormone leptin in MSCs from lupus patients [15, 100, 101]. In other cell types, the most robust data suggest paracrine senescence is mediated by the IGFBP family members [17, 75, 102–104] including the IGFBP-domain containing CCN1 (also known as IGFBP10) [105], cytokines CCL2 [18, 101], interleukin 1 beta (IL1β) [19], CXCL10 [106], transforming growth factor beta (TGFβ) [18, 19, 107, 108], the hormone prostaglandin E2 (PGE2) [109, 110], and extracellular vesicles [111]. IGFBP4 in particular has recently been suggested to mediate systemic spread of senescence: serum levels of IGFBP4 increase in response to DNA damage in humans and mice and IGFBP4 injections in mice lead to senescent cells accumulating in multiple organs, including among bone marrow MSCs [112]. We indeed found induction of IGFBP7 and CCL2 in MSCs during culture, and their rapid repression upon treatment with WNT agonists. These factors may therefore contribute to the WNT-mediated repression of paracrine senescence in MSCs. In basal breast cancer cells, activation of WNT/β-catenin signalling by WNT3A represses IGFBP5 (Liu et al. 2012); however, the mechanism has not been identified. Expression of IGFBPs is driven by IL6-STAT3 signalling [78] and NF-κB [113], and might thus respond to WNT signalling via NF-κB or IL6 repression. Careful analysis of the complex SASP factor network may therefore be required to assess the suitability of MSCs for therapeutic applications.

An uncontrolled paracrine spread of senescence would lead to loss of stem cells and regenerative capacity. Our findings can therefore have broader implications. The secretome of mesenchymal stromal cells in the bone marrow of aged mice induces senescence in other stromal cells, and inhibition of paracrine senescence using anti-inflammatory drugs reduces the number of senescent cells and improves bone formation [22]. Paracrine senescence is however not limited to mesenchymal cells: genetic induction of senescence in selected cells in the liver spreads to surrounding hepatocytes, resulting in liver fibrosis and impaired repair, and leading to pre-mature death in two murine liver injury models [107, 114]. WNT signals may therefore support stem cells in their niches or during regeneration not only by directly promoting their self-renewal, but also by preventing the induction of senescence-inducing factors in their environment. Since pro-inflammatory cytokines and TGFβ superfamily members are induced in response to tissue injury (reviewed in [115]), this mechanism might be particularly important during regenerative processes. Using WNT agonists to limit paracrine senescence after traumatic injury or genotoxic therapies may therefore provide an avenue to shield stem cell niches and encourage regeneration.

## Methods

### Detection of for senescence-associated lysosomal β-galactosidase

The percentage of senescent cells was determined by staining for senescence-associated lysosomal β-galactosidase using a modification of the protocol developed by [52]. Cells were washed twice in PBS, then fixed with a solution of 1% [v/v] formaldehyde (Sigma-Aldrich, Zwijndrecht, the Netherlands), and 0.5% glutaraldehyde [v/v] (Sigma-Aldrich) in PBS for 15 min at 4 °C and afterwards rinsed twice in distilled water. Subsequently, cells were incubated for 24 h at 37 °C with ca. 250 μl of staining solution per cm^2^ culture surface. The staining solution with a pH of 6.0 was made by dissolving per ml of distilled water 1 mg X-gal (5-bromo-4-chloro-3-indolyl-β-D-galactopyranoside) (Roche Diagnostics, Rotkreuz, Switzerland), 1.64 mg potassium hexacyanoferrate(III) (Sigma-Aldrich), 2.1 mg potassium hexacyanoferrate(II) trihydrate (Sigma-Aldrich), 2 μmol magnesium chloride hexahydrate (Sigma-Aldrich), 150 μmol sodium chloride, 7.3 μmol monohydrous citric acid (Sigma-Aldrich) and 25.3 μmol disbasic dodium phosphate dihydrate (Sigma-Aldrich). After incubation, the cells were rinsed twice in distilled water and either stained with 250 nM DAPI (4',6-diamidino-2-phenylindole) solution (Thermo-Fisher, Waltham, US) or counterstained using 1 g/l neutral red (Sigma-Aldrich) in a solution of 0.2% acetic acid in distilled water. Subsequently, the number of cells with cyan-coloured cytoplasm, indicating β-galactosidase activity, was counted, and plotted relatively to the total number of cells.

### MSC cell sourcing and culture

Human adult bone marrow-derived MSCs were isolated from femoral bone marrow aspirates of adults undergoing total hip replacement (MEC-2004–142 & MEC-2015–644; age: 43–88, given informed consent). Human paediatric MSCs were derived from left-over iliac crest bone chips of children undergoing palate cleft reconstruction (MEC-2014–16; 9–13 years, by implicit consent). The tissue utilized human tissue was procured as leftover/waste surgical material and it was reviewed and deemed exempt from full ethical review by the Erasmus MC Medical Ethical Committee under code MEC-2014–16. The protocols are in accordance with the ethical standards of our institution and with the 1964 Helsinki declaration and its later amendments or comparable ethical standards. Parents/guardians stated that they did not have any objection to the use of this tissue.

Bone chips were swirled twice in 10 ml expansion medium and the medium than plated in 175 cm^2^ culture flasks, whereas bone marrow aspirates were diluted with expansion medium to 20 ml and plated in 175 cm^2^ culture flasks. MSCs expansion medium consisted of: MEM-α (Gibco brand, Thermo-Fisher), containing 10% heat-inactivated FCS (Gibco brand, Thermo-Fisher), 50 μg/mL gentamicin (Invitrogen Life Technologies brand, Thermo-Fisher), 1.5 μg/ml amphotericin B [Fungizone™] (Invitrogen Life Technologies brand, Thermo-Fisher), 10^–4^ M L-ascorbic acid 2-phosphate (Sigma-Aldrich) and 1 ng/mL Fibroblast Growth Factor 2 [FGF2] (R&D Systems, Minneapolis, USA ems). After 24 h, the flasks were gently washed with PBS containing 1% FCS and adherent cells expanded in expansion medium, refreshed every three days, till ca. 80% confluence. Afterwards cells were passaged to 2300 cells/cm^2^ into expansion medium. When indicated, 250 ng/ml purified recombinant mouse WNT3A, made in house by genetically modified Schneider *Drosophila melanogaster* S2 cells or its vehicle 3-[(3-Cholamidopropyl)dimethylammonio]-1-propanesulfonate hydrate (CHAPS) (Sigma-Aldrich) were added to the medium. Medium was now refreshed daily, and cells passaged when reaching ca. 80% confluence. In all conditions, the expansion medium contained 1 ng/mL FGF2.

Adipose tissue-derived MSCs were isolated from human subcutaneous abdominal adipose obtained as waste material from female donors (age 46–52 years) with approval by the Medical Ethical Committee of the Erasmus MC (MEC-2014–092, by implicit consent). The adipose tissue was digested with collagenase type I (Gibco brand, Thermo-Fisher) for 1 h, then centrifuged and washed to remove adipocytes. Subsequently the pellet was suspended in Dulbecco’s Modified Eagle Medium with 1 g/l glucose (LG-DMEM; Gibco brand, Thermo-Fisher), filtered through a 100 μm strainer and plated in expansion medium. Upon 80% confluence, the cells were passaged and cultured under the same conditions as bone marrow-derived MSCs.

### EdU/CldU staining

To estimate cycling cells, cultures were treated with EdU for the indicated time points and then stained using the baseclick™ EdU-Click 488 kit (Sigma-Aldrich) according to manufacturer’s instructions. Cells were cultured on glass cover slips (18 mm ∅) for at least 24 h before beginning of the assay and seeded at a density so that at the end of the assay cells remained sub-confluent in the condition with the most rapid expansion. EdU was added to a final concentration of 10 µM to the medium. For > 24-h EdU pulses in experiments with daily refreshment, EdU was added to the fresh medium to be added to the cells. If the EdU pulse was below 24 h or in experiments on conditioned medium effects, where refreshment was not daily, the EdU was added to the medium already on the cells so that the next refreshment would coincide with cell harvest. After the indicated time points, the medium was aspirated, and the cells washed in PBS. Cells were then fixed with 3.7% formaldehyde, washed with PBS containing 3% BSA (Sigma-Aldrich) [wash solution] and, permeabilized with 0.5% Triton X-100 in wash solution. The coverslips were then placed onto a paraffin film (Bemis, Neenah, USA) and exposed to 50 µl of reaction cocktail (consisting of deionized water, reaction buffer, catalyst solution, fluorophore 6-FAM-azide and buffer additive as per manufacturer protocol) and incubated at room temperature in a dark for 30 min. Subsequently, the coverslips were rinsed in wash solution and anti-body-based stainings were now performed as indicated below. The coverslips were rinsed in distilled water and stained with a 250 nM DAPI solution for 5 min. Coverslips were mounted onto slides in a mounting solution (90% glycerol (Sigma-Aldrich), 10% PBS, 50 μg/ml gentamycin,1.5 μg/ml amphotericin B) and affixed using non-fluorescent nail polish.

To identify cells exiting cell cycle, cells were first labelled with 10 µM CldU for 24 h, then the medium aspirated and the wells washed thrice with PBS and subsequently cultured with 10 µM EdU for further 24 h. The cells were then first stained using the baseclick™ EdU-Click 488 kit as described above, then stained using a rat-anti-BrDU antibody also recognizing CldU (BU1/75, Biotechne, Abingdon, UK), washed twice with PBS and stained with a goat-anti-rat Alexa594-tagged antibody (Abcam, Cambridge, UK). To denature the DNA for CldU staining, a treatment with 2 M HCl (Sigma-Aldrich) for 60 min at 37 °C was inserted into EdU staining protocol after fixation but before permeabilization. We verified that denaturation had no effect on EdU staining quality, that the EdU detection kit did not stain CldU-only labelled cells and that staining of EdU-only labelled cells with the CldU antibody was minimal.

### Immunocytochemistry

Cells were cultured on coverslips, then fixed and permeabilized as described for the EdU staining. Subsequently, cells were stained with either 2.5 µg/ml mouse-anti-Ki67 (Clone B56; BD Pharmingen, Franklin Lakes, USA) antibody or 1ug/ml mouse-anti-γH2AX [phospho S139] antibody (clone JBW30; Millipore brand, Sigma-Aldrich) in PBS with 3% BSA for 2 h, rinsed twice in PBS with 0.5% Triton X-100, then stained with a goat-anti-mouse Alexa594-tagged antibody (Abcam) for 1 h and rinsed twice with twice in PBS with 0.5% Triton X-100. Coverslips were washed in deionized water, stained DAPI and mounted as indicated above. The positive control for γH2AX staining were MSCs irradiated with 15 Gy using a gamma source (Gammacell; Nordion, Abingdon, UK) and fixed 15 min later.

### Real-time PCR

RNA was isolated using 0.1 ml phenol/guanidine thiocyanate solution (TriPure Isolation Reagent; Sigma-Aldrich) per cm^2^ culture surface with 5 µg/ml glycogen (Roche Diagnostics) added to improve yield. The solution of lysed cells was then homogenized by pipetting, incubated for 15 min at RT, and RNA isolated using a chloroform–ethanol extraction according to the TriPure Isolation Reagent protocol. Subsequently, the concentration of RNA was estimated using a spectrophotometer (NanoDrop 8000; Isogen Life Science B.V, De Meern, the Netherlands) at 260 and 280 nm and the RNA treated with amplification grade deoxyribonuclease I (Invitrogen brand, Thermo-Fisher) to remove DNA. The SuperScript™ II Reverse Transcriptase kit (Invitrogen brand, Thermo-Fisher) was used according to manufacturer’s instructions to synthetize cDNA, using Oligo(dT)s and a purified dNTP mix (Thermo-Fisher). Real-time PCR reactions were run using the platinum taq DNA polymerase kit (Invitrogen brand, Thermo-Fisher) according to manufacturer’s instructions on a combined thermal cycler/detection system (CFX96Touch, Biorad, Hercules, USA) using SYBR-Green (Thermo-Fisher) to quantify nucleic acid concentration. Primers were picked from the PrimerBank database (https://pga.mgh.harvard.edu/primerbank/) [116] and validated in house.

Table with Primers.

**Table:** 

Gene	PrimerBank ID	Forward Primer	Reverse Primer
GAPDH	378404907c1	GGAGCGAGATCCCTCCAAAAT	GGCTGTTGTCATACTTCTCATGG
ACTB	312176409c1	ACCGGGCATAGTGGTTGGA	ATGGTACACGGTTCTCAACATC
HPRT1	164518913c1	CCTGGCGTCGTGATTAGTGAT	AGACGTTCAGTCCTGTCCATAA
AXIN2	195927058c1	CAACACCAGGCGGAACGAA	GCCCAATAAGGAGTGTAAGGACT
TERT	301129199c1	AAATGCGGCCCCTGTTTCT	CAGTGCGTCTTGAGGAGCA
IL6	224831235c1	ACTCACCTCTTCAGAACGAATTG	CCATCTTTGGAAGGTTCAGGTTG
IGFBP4	10835021a1	GGTGACCACCCCAACAACAG	GAATTTTGGCGAAGTGCTTCTG
IGFBP5	171460920c1	ACCTGAGATGAGACAGGAGTC	GTAGAATCCTTTGCGGTCACAA
IGFBP7	359465607c1	CGAGCAAGGTCCTTCCATAGT	GGTGTCGGGATTCCGATGAC
CDKN1A	310832423c1	TGTCCGTCAGAACCCATGC	AAAGTCGAAGTTCCATCGCTC
CCL2	4506841a1	CAGCCAGATGCAATCAATGCC	TGGAATCCTGAACCCACTTCT

### IL6 detection by ELISA

IL6 protein concentration was measured in medium conditioned by senescent cells for 24 h using a solid-phase sandwich ELISA for human IL6 (DY206-05, R&D brand, Biotechne) according to manufacturer’s instructions with three technical replicates per biological sample. Serial dilutions of human recombinant IL-6 standard were included in each assay to obtain a standard curve. Absorbance was measured at a wavelength of 450 nm with wavelength correction set at 650 nm using a microplate reader (Bio-Rad Laboratories).

### Transcriptome analysis

Bone marrow aspirates from three post-menopausal women (ages 64, 64, 73) were plated as described above and cultured for nine days in expansion medium. For transcriptome analysis (see scheme Fig. 1B), cells isolated from each donor were passaged into expansion medium without additional components (10,000 cells/cm^2^), with vehicle CHAPS (2300 cells/cm^2^) or with 250 ng/ml WNT3A (2300 cells/cm^2^). After 24 h, the flasks without additional components were switched to expansion medium with FGF2 and with CHAPS (+ vehicle) or with FGF2 and with WNT3A (+ WNT3A). After six hours, these cells (passage 1 samples) were harvested, lysed in TriPure Isolation Reagent, and RNA isolated as described above, but without addition of glycogen. Cells in the remaining two flasks were expanded with daily refreshing of the expansion medium with CHAPS or WNT3A, respectively, until 80% confluence was reached and then passaged to 2300 cells/cm^2^. MSCs were expanded in this way until expansion ceased. At passage four, WNT3A and CHAPS expanded cells were plated (10,000 cells/cm^2^); after 24 h, the medium was refreshed and 6 h later, the cells (passage 4 samples) lysed in TriPure Isolation Reagent.

RNA sequencing library was prepared using the Trueseq kit (Illumina, Eindhoven, the Netherlands). Average amount of paired reads per sample were 30 million and sequencing was performed with an Illumina HiSeq 2000 (Illumina). Data were aligned (paired-end read, inner-mate distance 50 bp) to refseq human genome hg19 using TopHat & Bowtie2. Cufflinks was used to generate gene expression levels, the UCSC refseq annotation for hg19 reference data was used as reference set and transcripts and genes were counted.

To filter transcripts with no or very low expression, only transcripts having at least 20 counts in total over all conditions were considered for further analysis. Differentially expressed genes were then determined using the *R* package DESeq2 for the comparisons of passage one to passage four MSCs in vehicle (referred to as change over time in culture) as well as vehicle to WNT3A cultured MSCs both at passage one (six hours of treatment) and at passage four (continuous treatment passage one to four) [117]. A list of all transcripts with their fold change as well as the adjusted (Benjamini–Hochberg/false discovery rate (FDR)) *p* values for the aforementioned comparisons is given in supplementary file 1. The annotated R-script used to determine differential gene expression, pathway enrichment and gene set enrichment as well as create the heatmaps shown in Figs. 1 and 4 is provided as supplementary file 4.

### Pathway analysis

Changes in pathway activity over passage in presence or absence of WNT3A were determined by selecting genes up- or down-regulated at least twofold and with a adjusted *p *values of < 0.05 and using the *R* packages clusterProfiler and ReactomePA [118, 119] with annotation derived from the Reactome pathway database to identify significantly changes pathways (*cut-off: adjusted (Benjamini–Hochberg/FDR) p* < *0.001*) [120]. Pathways and genes associated with these pathways as well as the according statistical parameters of enrichment are provided in supplementary file 2.

### Gene set enrichment analysis

Gene set enrichment analysis according to [121] was performed for the indicated five gene sets on the 20.000 genes with the highest average expression over all conditions. The data were processed using the R package clusterProfiler [122], employing the FGSEA method [123] and using the Holm–Bonferroni method for multiple test correction. The paracrine senescence response gene set is based on microarray data from [18] comparing gene expression between IMR90 human fibroblasts exposed to the secretome of senescent IMR90s (treated) to that of IMR90s exposed to the secretome of non-senescent IMR90s (control). We generated the gene set by selecting all genes that were significantly (*p* < 0.0001) upregulated more than ten-fold in treated over control cells. The WNT/beta-catenin signalling pathway gene sets were sourced via the Molecular Signatures Database (UC San Diego and Broad Institute) [121, 124] and are listed below. The other gene sets are based on literature (SASP [10], paracrine senescence [18], cell cycle [44], DNA repair [45], quiescence vs. senescence [56, 125], senescence up- and down-regulated genes [48, 49]) and for convenience are provided as tables in supplementary file 3. Random gene sets were generated from a pool of all human genes (GRCh38.p13) as control matching the set size of the largest gene set analysed in an individual comparison.

**Table:** 

WNT/beta-catenin gene sets			
ID in figure	Full Name	MSigDB ID	Source
GOBP	Gene Ontology biological process: canonical WNT signalling	M12752	GO: 0,060,070
HALLMARK	Molecular Signatures Database Hallmark Genesets: WNT/beta-catenin signalling	M5895	MSigDB: M5895
KEGG	Kyoto Encyclopedia of Genes and Genomes: WNT signalling pathway	M19428	KEGG: hsa04310

### Paracrine senescence studies using conditioned medium

Cells were rendered senescent either by irradiation with 80 Gray in a RS320 X-Ray machine (X-Strahl, Camberley, UK) or by culture until expansion had ceased (defined as the cell population not increasing over seven days). After irradiation, cells were cultured for at least seven days to allow senescence to occur. Senescent cells were then seeded at 20,000 cells/cm^2^ with 0.2 ml/cm^2^ expansion medium and cultured at least for 24 h before treatment. When comparing non-senescent to senescent cells, seeding cell density was adjusted to ensure all conditions contained approximately equal cell densities at the end of treatment, which was confirmed by cell count after medium harvest.

The cells were treated with 250 ng/ml WNT3A, 12 µM BAY11-7082 (BAY) (Santa-Cruz, Heidelberg, Germany), 1 µM dexamethasone (Sigma-Aldrich), 3 µM CHIR99021 (CHIR) (Stem Cell Technologies, Cologne, Germany), 10 ug/ml IL6-neutralizing antibody (2.11 B12) (kind gift by Frank Grosveld, Harbour Antibodies/Erasmus MC) or with the equivalent amounts of the according vehicle: CHAPS (for WNT3A), DMSO (BAY, CHIR), ethanol (dexamethasone) or PBS (IL6-neutralizing antibody). We optimized treatment duration for the different compounds—to ensure equal culture duration for cells, all conditions were cultured for 6 days, with conditions with shorter treatment duration cultured with expansion medium prior treatment so that all treatments ended on the sixth day. Treatment duration were: 6 days for WNT3A and CHIR and 3 days for BAY and dexamethasone. Non-neutralizing IL6 antibodies of the same isotype were used as controls for the neutralizing IL6 antibody. In all conditions, including pre-culture in expansion medium, refreshment was daily. At the end of treatment, cells were washed twice gently with PBS and refreshed with 0.2 ml/cm^2^ expansion medium and a control flask without cells refreshed with 0.2 ml/cm^2^ expansion medium prepared. After 24 h, the conditioned medium was harvested, centrifuged at 300 g for 8 min and the supernatant stored. We found no difference between fresh and frozen (− 80 °C) conditioned and control medium regarding effects on proliferation and number of β-galactosidase-positive cells, therefore the supernatants were stored at  −  80 °C for no more than four weeks. As control, unconditioned medium was made by incubating expansion medium for 24 h in a cell-free culture plate at 37 °C, centrifuging at 300 g for 8 min and storing the supernatant at − 80 °C.

Recipient cells were plated at 2,300 cells/cm^2^ and cultured for 24 h in expansion medium before being exposed to conditioned or control medium with refreshment every second day. Condition and control media were used undiluted, except in the dosage titration experiment where the two were mixed at the indicated ratios. The recipient cell cultures for all conditions were processed (analysed or passaged) simultaneously, either after the indicated time spans or when the first condition reached ca. 80% confluence (this was usually the cells cultured in unconditioned medium after 5–7 days). In the experiment where duration of exposure to conditioned medium was analysed, recipient cells were first treated for one, three or six days, with culture switching to control medium for the remaining time till 6 days. When expansion over multiple passages was analysed, recipient cell number was counted, and all conditions passaged again to 2300 cells/cm^2^ into the according media.

### Statistical analysis and data processing

Counting of cells for assays (γH2AX, β-galactosidase, EdU, CldU, Ki67) was performed blinded by at least one experimenter (Johannes Lehmann, Natasja Franceschini, Danai Chatzivasileiou). For expansion assays, at least four samples were taken and counted per replicate to determine cell number. Statistical tests were run and data plotted using R, foremost using the ggplot2 package [126, 127]. Comparisons between groups were made using the Wilcoxon–Mann–Whitney test (two-tailed, samples not paired unless otherwise indicated), correlation was assessed based on Spearman’s rank correlation coefficient. Number of replicates are given in the figure legends, when wells are indicated these are independent cultures derived from one donor, otherwise the number of donors is given.

## Supplementary Information

Below is the link to the electronic supplementary material.

Supplementary file1 (XLSX 8005 KB)

Supplementary file2 (XLSX 585 KB)

Supplementary file3 (XLSX 19 KB)

Supplementary file5 (PDF 200 KB)

Supplementary file4 (DOCX 2821 KB)

## Data Availability

The transcriptomics raw and processed data have been deposited in NCBI's Gene Expression Omnibus and are accessible through GEO Series accession number GSE152112. Differentially expressed genes, enriched pathways, custom gene sets derived from literature and the analysis pipeline (annotated R script) have been made available as supplementary files 1–4.
