# Supplementary material for: WNT/beta-catenin signalling interrupts a senescence-induction cascade in human mesenchymal stem cells that restricts their expansion
Source: Cell Mol Life Sci. 2022 Jan 20;79(2):82. doi: 10.1007/s00018-021-04035-x (PMC8770385; doi:10.1007/s00018-021-04035-x)
Supplement: Supplementary file 4 — Supplementary file5 (PDF 200 KB) [file 18_2021_4035_MOESM4_ESM.pdf]

MAN_SAS_RNA_Version2021_V5

Johannes Lehmann

15/10/2021

Install and load required packages

```
#Bioconductor Manager for further installations
if(FALSE){install.packages("BiocManager")
  library(BiocManager)}
#for GSEA
if(FALSE){
  BiocManager::install("clusterProfiler")
  BiocManager::install("fgsea")
  BiocManager::install("DESeq2")
  BiocManager::install("ReactomePA")}
#for annotation
if(FALSE){BiocManager::install("org.Hs.eg.db")}

#for enrichment plots
if(FALSE){
require(devtools)
devtools::install_github("YuLab-SMU/enrichplot")
remotes::install_github("YuLab-SMU/ggtree")
install.packages("ggstar")
install.packages("ggnewscale")
##install old version of dplyr due to conflict with ggtree/treeplot
install_version("dplyr", version = "1.0.5", repos = "http://cran.us.r-project.org")}

#general purpose plotting and processing
if(FALSE){
  install.packages(c("tidyverse",
                     "readxl", "readr","pheatmap","ggplot2","scales","gbeeswarm",
                     "patchwork","RColorBrewer","svglite","Cairo","here"))}
```

Load required packages

```
#enrichment analysis
library(DOSE)
library(ReactomePA)

#GSEA
library(clusterProfiler)

library(fgsea)
#displaying enrichment
library(enrichplot)
library(ggstar)
library(ggnewscale)
#general packages
library(dplyr)

library(readxl)
library(tidyverse)

library(stringr)
library(pheatmap)
library(ggplot2)
library(scales)
```

```
library(ggbeeswarm)
library(patchwork)
library(readr)

# Load library for DESeq2
library(DESeq2)
library(RColorBrewer)
#for export of graphs in svg format
library(svglite)
#for export of graphs in png format
library(Cairo)
#for file finding
library(here)

#for annotation conversion
library(org.Hs.eg.db)

#check if BiocManager packages are up-to-date (if too many oudated packages, errors will result in the next steps)
BiocManager::valid()

#theme for plotting
theme_white_on_black <- function(){
  theme_classic(base_line_size = 1, base_family="Arial", base_size = 8)+
    theme(panel.background = element_rect(fill = "black", colour = "black"))+
    theme(plot.background = element_rect(fill = "black", colour = "black"))+
    theme(axis.line = element_line(size = 1, colour = "white"))+
    theme(axis.ticks = element_line(size = 1, colour = "white"))+
    theme(axis.title = element_text(family = "Arial", size = 8, colour = "white"))+
    theme(axis.text = element_text(family = "Arial", size = 8, colour = "white"))+
    theme(legend.background = element_rect(fill = "black", colour = "black"))+
    theme(legend.text = element_text(family = "Arial", size = 8, colour = "white"))+
    theme(legend.title = element_text(family = "Arial", size = 8, colour = "white"))+
    theme(strip.background = element_rect(fill = "black", colour = "black"))+
    theme(strip.text = element_text(family = "Arial", size = 8, colour = "white"))}
theme_black_on_white <- function(){
  theme_classic(base_line_size = 1, base_family="Arial", base_size = 8)+
    theme(panel.background = element_rect(fill = "white", colour = "white"))+
    theme(plot.background = element_rect(fill = "white", colour = "white"))+
    theme(axis.line = element_line(size = 1, colour = "black"))+
    theme(axis.ticks = element_line(size = 1, colour = "black"))+
    theme(axis.title = element_text(family = "Arial", size = 8, colour = "black"))+
    theme(axis.text = element_text(family = "Arial", size = 8, colour = "black"))+
    theme(legend.background = element_rect(fill = "white", colour = "white"))+
    theme(legend.text = element_text(family = "Arial", size = 8, colour = "black"))+
    theme(legend.title = element_text(family = "Arial", size = 8, colour = "black"))+
    theme(strip.background = element_rect(fill = "white", colour = "white"))+
    theme(strip.text = element_text(family = "Arial", size = 8, colour = "black"))+
    theme(text = element_text(family = "Arial"))}

#Here we choose a theme
theme_set(theme_black_on_white())
```

Import sequencing data

```
#where are the files
##sets path to where this file/project is located
here()
<…>

#reading in sequencing data
##numbers should be integers for DESeq analysis
RNA_integer <- read_csv("MAN_RNAseq_from_JE2 Count Data V04_V1_integers_wsums.csv",
                        col_types = cols(entrezgene = col_number(),
```

```
                                    FC_P1_R403 = col_integer(), FC_P1_R287 = col_integer(),
                                    FC_P1_D079 = col_integer(), FW_P1_R403 = col_integer(),
                                    FW_P1_R287 = col_integer(), FW_P1_D079 = col_integer(),
                                    FC_P4_R403 = col_integer(), FC_P4_R287 = col_integer(),
                                    FC_P4_D079 = col_integer(), FW_P4_R403 = col_integer(),
                                    FW_P4_R287 = col_integer(), FW_P4_D079 = col_integer(),
                                    rowsums = col_integer()))

#get sample names from columns
samples_ID <- colnames(RNA_integer[,c(4:15)])

#for DESeq2
##filter low expressed genes
##time consuming step due to inefficient sum calculation, ran outside of script and encoded as rowsums column
if(FALSE){RNA_forDSEQ <- RNA_integer[,c(1,4:15)] %>% rowwise() %>% mutate(counts_sum = sum(c_across(2:13)))
%>%
  ungroup()}
##remove duplicates and turn into rowname format
RNA_forDSEQ <- RNA_integer %>% filter(rowsums > 20) %>% dplyr::select(1,4:15) %>% filter(duplicated(query) == FAL
SE) %>%
    column_to_rownames(var = "query")
#save file to speed up later processing when continuing from here
saveRDS(RNA_forDSEQ, file = "RNA_forDSEQ.rds")
```

determine differentially expressed genes

```
#load saved R file (if above chunks are not re-run)
RNA_forDSEQ <- readRDS(file = "RNA_forDSEQ.rds")
#what sample types do we have?
colnames(RNA_forDSEQ)

##  [1] "FC_P1_R403" "FC_P1_R287" "FC_P1_D079" "FW_P1_R403" "FW_P1_R287"
##  [6] "FW_P1_D079" "FC_P4_R403" "FC_P4_R287" "FC_P4_D079" "FW_P4_R403"
## [11] "FW_P4_R287" "FW_P4_D079"

#create metadata
##dataframe with row order matching column order of sequencing data to be processed
if(TRUE){
  RNA_meta <- data.frame(ID = samples_ID,
                      status = as.factor(c("vehicle_P1","vehicle_P1","vehicle_P1","WNT_P1","WNT_P1","WNT_P1",
                                    "vehicle_P4","vehicle_P4","vehicle_P4","WNT_P4","WNT_P4","WNT_P4")),
                      donor = as.factor(c("R403","R287","D079","R403","R287","D079",
                                   "R403","R287","D079","R403","R287","D079")))   %>%
    column_to_rownames(var = "ID") }

##check if the row names and column names for metadata and sequencing data match
all(rownames(RNA_meta) == colnames(RNA_forDSEQ))

## [1] TRUE

match(rownames(RNA_meta), colnames(RNA_forDSEQ))

##  [1]  1  2  3  4  5  6  7  8  9 10 11 12

#DESeq2 analysis
#I adapted the pipeline below from https://avikarn.com/2020-07-02-RNAseq_DeSeq2/ and from https://workshop.eupathd
b.org/bop/pdfs/beginner_DeSeq2.pdf
#create DESeq2 object
dds <- DESeqDataSetFromMatrix(countData = RNA_forDSEQ,
                           colData = RNA_meta,
                           design = ~ donor + status)
dds <- estimateSizeFactors(dds)
```

```
#save file to speed up later processing when continuing from here
saveRDS(dds, file = "DESeqDataSet.rds")

dds_normalzd <- counts(dds, normalized=T)

#extract differentially expressed genes
difex  <- DESeq(dds)

## using pre-existing size factors

## estimating dispersions

## gene-wise dispersion estimates

## mean-dispersion relationship

## final dispersion estimates

## fitting model and testing

resultsNames(difex)

## [1] "Intercept"                        "donor_R287_vs_D079"
## [3] "donor_R403_vs_D079"               "status_vehicle_P4_vs_vehicle_P1"
## [5] "status_WNT_P1_vs_vehicle_P1"     "status_WNT_P4_vs_vehicle_P1"

##WNT = with WNT3A at P4
##tim = over time (passage) from P1 to P4 in vehicle
res_tim <-results(difex, contrast = c("status","vehicle_P4","vehicle_P1"))
res_WNT <-results(difex, contrast = c("status","WNT_P4","vehicle_P4"))
res_WNT_P1 <-results(difex, contrast = c("status","vehicle_P1","WNT_P1"))

#generate summaries of results
summary(res_tim)

##
## out of 21933 with nonzero total read count
## adjusted p-value < 0.1
## LFC > 0 (up)       : 1722, 7.9%
## LFC < 0 (down)     : 1585, 7.2%
## outliers [1]       : 0, 0%
## low counts [2]     : 5528, 25%
## (mean count < 9)
## [1] see 'cooksCutoff' argument of ?results
## [2] see 'independentFiltering' argument of ?results

summary(res_WNT)

##
## out of 21933 with nonzero total read count
## adjusted p-value < 0.1
## LFC > 0 (up)       : 2960, 13%
## LFC < 0 (down)     : 3235, 15%
## outliers [1]       : 0, 0%
## low counts [2]     : 2977, 14%
## (mean count < 4)
## [1] see 'cooksCutoff' argument of ?results
## [2] see 'independentFiltering' argument of ?results

summary(res_WNT_P1)

##
## out of 21933 with nonzero total read count
## adjusted p-value < 0.1
```

```
## LFC > 0 (up)       : 17, 0.078%
## LFC < 0 (down)     : 98, 0.45%
## outliers [1]       : 0, 0%
## low counts [2]     : 5103, 23%
## (mean count < 7)
## [1] see 'cooksCutoff' argument of ?results
## [2] see 'independentFiltering' argument of ?results
```

pathway enrichment analysis

```
#add gene name annotations (already in original file)
res_tim_df <- as.data.frame(res_tim[order(res_tim$pvalue),]) %>% rownames_to_column(var = "ENSG")
res_WNT_df <- as.data.frame(res_WNT[order(res_WNT$pvalue),]) %>% rownames_to_column(var = "ENSG")
res_WNT_P1_df <- as.data.frame(res_WNT_P1[order(res_WNT_P1$pvalue),]) %>% rownames_to_column(var = "ENSG
")
#what annotations are available
columns(org.Hs.eg.db)

##  [1] "ACCNUM"       "ALIAS"        "ENSEMBL"      "ENSEMBLPROT"  "ENSEMBLTRANS"
##  [6] "ENTREZID"     "ENZYME"       "EVIDENCE"     "EVIDENCEALL"  "GENENAME"
## [11] "GENETYPE"     "GO"           "GOALL"        "IPI"          "MAP"
## [16] "OMIM"         "ONTOLOGY"     "ONTOLOGYALL"  "PATH"         "PFAM"
## [21] "PMID"         "PROSITE"      "REFSEQ"       "SYMBOL"       "UCSCKG"
## [26] "UNIPROT"

#annotate
ID_lookuptable <- AnnotationDbi::select(org.Hs.eg.db, keys = RNA_integer$query,
                                        columns=c("ENSEMBL", "SYMBOL","GENENAME", "ENTREZID"), keytype="ENSEMBL")

## 'select()' returned many:many mapping between keys and columns

                                ##if only genes with symbols needed then add (but enrichment takes entrez)
                                #filter(is.na(SYMBOL) == FALSE)

#unite data in dataframe for convenient viewing
RNA_sig <- RNA_forDSEQ %>% rownames_to_column(var = "ENSG") %>%
  inner_join(ID_lookuptable, by = c("ENSG" = "ENSEMBL")) %>%
  inner_join(res_tim_df, by = "ENSG") %>% inner_join(res_WNT_df, by = "ENSG", suffix = c("_tim","_WNT")) %>%
  filter(duplicated(SYMBOL) == FALSE)

#add annotations to simple one contrast dataframes
res_tim_anot <- res_tim_df %>% inner_join(ID_lookuptable, by = c("ENSG" = "ENSEMBL"))
res_WNT_anot <- res_WNT_df %>% inner_join(ID_lookuptable, by = c("ENSG" = "ENSEMBL"))
res_WNT_P1_anot <- res_WNT_P1_df %>% inner_join(ID_lookuptable, by = c("ENSG" = "ENSEMBL"))

#export one-contrast dataframes as csv for supplement
write.csv(res_tim_anot, file = "DESeq2_time_vehicle_P1vsP4.csv")
write.csv(res_WNT_anot, file = "DESeq2_WNTvsVehicle_P4.csv")
write.csv(res_WNT_P1_anot, file = "DESeq2_WNTvsVehicle_P1.csv")




#extract significantly up- or downregulated genes
##WNT = with WNT3A at P4
##tim = over time (passage) from P1 to P4 in vehicle
sig_tim_up <- res_tim_anot %>% filter(log2FoldChange > 0.69, padj < 0.05) %>% arrange(desc(log2FoldChange)) %>%
pull(ENTREZID)
sig_tim_dn <- res_tim_anot %>% filter(log2FoldChange < -0.69, padj < 0.05) %>% arrange((log2FoldChange)) %>% pull(
ENTREZID)
sig_WNT_up <- res_WNT_anot %>% filter(log2FoldChange > 0.69, padj < 0.05) %>% arrange(desc(log2FoldChange)) %
>% pull(ENTREZID)
```

```
sig_WNT_dn <- res_WNT_anot %>% filter(log2FoldChange < -0.69, padj < 0.05) %>% arrange((log2FoldChange)) %>%
pull(ENTREZID)

#for cross-comparison make matrix
RNA_cross <- RNA_sig %>% dplyr::select(SYMBOL,ENTREZID,log2FoldChange_tim, padj_tim, log2FoldChange_WNT,
padj_WNT) %>%
  mutate(WNT = case_when((log2FoldChange_WNT > 0.69 & padj_WNT < 0.05) ~ "WNT_up",
                         (log2FoldChange_WNT < -0.69 & padj_WNT < 0.05) ~ "WNT_dn",
                         ((log2FoldChange_WNT <= 0.69 & log2FoldChange_WNT >= -0.69) |
                            (padj_WNT >= 0.05 | is.na(padj_WNT)) ~ "WNT_NC")),
        TIM = case_when((log2FoldChange_tim > 0.69 & padj_tim < 0.05) ~ "tim_up",
                         (log2FoldChange_tim < -0.69 & padj_tim < 0.05) ~ "tim_dn",
                         ((log2FoldChange_tim <= 0.69 & log2FoldChange_tim >= -0.69) |
                            (padj_tim >= 0.05 | is.na(padj_tim)) ~ "tim_NC"))) %>%
  ##remove genes that are not changed either with WNT or with time
  ##only keep genes that change UP with WNT and DOWN with time or DOWN with WNT and UP with time
  filter(TIM != "tim_NC" & WNT != "WNT_NC") %>% filter((WNT == "WNT_up" & TIM == "tim_dn") |(WNT == "WNT_dn" &
TIM == "tim_up"))

#combination
sig_tim_dn_WNT_up <- RNA_cross %>% filter(WNT == "WNT_up" & TIM == "tim_dn") %>% pull(ENTREZID)
sig_tim_up_WNT_dn <- RNA_cross %>% filter(WNT == "WNT_dn" & TIM == "tim_up") %>% pull(ENTREZID)
#background genes (all genes in this dataset after count filtering) to be used as reference background
background_genes <- RNA_sig$ENTREZID

#create function for enrichment analysis, processing, plotting and saving
enrichplotter <- function(genevector){
#generate name for file from input
genevector_name <- deparse(substitute(genevector))
labelling_csv <- paste("enrichment", "_for_", genevector_name, ".csv", sep = "")
labelling_pfilt_csv <- paste("enrichment_filter-p001", "_for_", genevector_name, ".csv", sep = "")
labelling_svg <- paste("enrichment", "_for_", genevector_name, ".svg", sep = "")
#enrichment analysis
reactome <- enrichPathway(gene = genevector,
              pvalueCutoff = 1, qvalueCutoff = 1, pAdjustMethod = "BH",
              organism = "human", #minGSSize = 10,#maxGSSize = 600,
              universe = background_genes,
              readable=TRUE)

#order results and
reactome_all <- reactome %>%
  mutate(richFactor = Count / as.numeric(sub("/\\d+", "", BgRatio))) %>%
  mutate(geneRatio = parse_ratio(GeneRatio), bgRatio = parse_ratio(BgRatio)) %>% arrange(desc(geneRatio))
reactome_order <- reactome_all %>% filter(p.adjust < 0.001)

#extract as dataframe
##can be used for easier viewing of all enriched pathways
##save as csv for reproducibility (plots limit which pathways are shown by cut-off)
reactome_df <- data.frame(reactome_all@result)
reactome_order_df <- data.frame(reactome_order@result)
write.csv(reactome_df, file = labelling_csv)
write.csv(reactome_order_df, file = labelling_pfilt_csv)
#generate plot
plot.reactome <- enrichplot::dotplot(reactome_order, showCategory = 8, font.size = 8)

#save as svg for Adobe Illustrator processing
svglite(filename = labelling_svg, width = 4, height = 4, pointsize = 8)
plot.reactome
##do not call dev.off(), seems to corrupt svg files when running function repeatedly?
#return plot as function outcome
return(plot.reactome)
}
```

```
#analyse genelists for enrichment using above function
enrichplotter(sig_tim_up_WNT_dn)
dev.off()

enrichplotter(sig_tim_dn_WNT_up)
dev.off()

enrichplotter(sig_tim_up)
dev.off()

enrichplotter(sig_tim_dn)
dev.off()

enrichplotter(sig_WNT_up)
dev.off()

enrichplotter(sig_WNT_dn)
dev.off()

#for overview graph
if(FALSE){
patch <- enrichplotter(sig_tim_up_WNT_dn) + enrichplotter(sig_tim_dn_WNT_up) +
  enrichplotter(sig_tim_up) + enrichplotter(sig_tim_dn) +
enrichplotter(sig_WNT_up) + enrichplotter(sig_WNT_dn) + plot_layout(ncol = 2, nrow = 3)
svglite(filename = "overview.svg", width = 10, height = 15, pointsize = 8)
patch
dev.off()}
```

#Heatmaps

```
#import gene lists
COP <- read_excel("MAN_SASP pathway.xlsx",
                 sheet = "Coppe2")
COP_labelled <- mutate(COP, type_lab = case_when(type == 1 ~ "interleukins",
                                                type == 2 ~ "chemokines",
                                                type == 3 ~ "growth factors & IGFBPs",
                                                type == 4 ~ "MMPs & protease interactome",
                                                type == 5 ~ "shed receptors"))
ACOSTA <- read_csv("Acosta_microarray_paracrine_induced2.csv")

REPAIR <- read_excel("MAN_RNAseq_GeneLists_V1.xlsx",
                   sheet = "Repair - Wood (symbols) (red)")
CYCLE <- read_excel("MAN_RNAseq_GeneLists_V1.xlsx",
                   sheet = "Cycle - Giotti (symbols)")



#convert into tidy dataset
RNA_tidy <- RNA_sig %>% dplyr::select(SYMBOL, one_of(samples_ID)) %>% pivot_longer(names_to = "ID", values_to =
"counts", cols = one_of(samples_ID)) %>%  separate(ID, into = c("treatment","passage","donor"), sep = "_")

#calculate means to reduce heatmap columns
#pivot wider to allow heatmap row conversion
##use select to choose order of columns in heatmap
RNA_means <- RNA_tidy %>% group_by(SYMBOL, treatment, passage) %>% summarise(counts_mean = mean(counts)
) %>% pivot_wider(names_from = c("passage","treatment"),values_from = counts_mean) %>% dplyr::select(SYMBOL, P1
_FC, P1_FW, P4_FC, P4_FW)
```

`summarise()` has grouped output by 'SYMBOL', 'treatment'. You can override using the `.groups` argument.

```
#extract only genes of interest for heatmap
##convert from tidy into rownamed dataframe
RNA_SASP <- RNA_means %>% filter(SYMBOL %in% COP$symbol) %>%
```

```
  column_to_rownames(var = "SYMBOL")
RNA_CYCLE <- RNA_means %>% filter(SYMBOL %in% CYCLE$symbol) %>%
  column_to_rownames(var = "SYMBOL")
RNA_REPAIR <- RNA_means %>% filter(SYMBOL %in% REPAIR$symbol) %>%
  column_to_rownames(var = "SYMBOL")

#fuse Acosta for ordering
RNA_order_ACOSTA <- ACOSTA %>% inner_join(RNA_means, by = c("GeneSymbol" = "SYMBOL")) %>% filter(is.na(GeneSymbol) == FALSE) %>% filter(duplicated(GeneSymbol) == FALSE) %>% filter((logFC.4 > 2.3) & adj.P.Val.4 < 0.0001)
write_csv(RNA_order_ACOSTA, file = "paracrine_acosta_filtered.csv")
RNA_ACOSTA <- RNA_order_ACOSTA %>% dplyr::select(GeneSymbol, P1_FC, P1_FW, P4_FC, P4_FW) %>% column_to_rownames(var = "GeneSymbol")
RNA_ACOSTA_annot <- RNA_order_ACOSTA %>% dplyr::select(GeneSymbol, logFC.4) %>% column_to_rownames(var = "GeneSymbol")

#annotate SASP types based on Coppe paper
COP_annotation <- COP_labelled %>% dplyr::select(symbol, type_lab) %>% column_to_rownames(var = "symbol")
ann_colors = list(type_lab = c("interleukins" = "gold",
             "chemokines" = "orange",
             "growth factors & IGFBPs" = "dark red",
             "MMPs & protease interactome" = "dark cyan",
             "shed receptors" = "purple"
             ))
#Acosta colours
library(RColorBrewer)
ann_ACOSTA <- list(logFC.4 = color_palette(c("dark cyan","gold")))

#heatmap function
pheatmap_def <- function(RNA_set, clusternon, annotation_file, annotation_colors){
set_name <- deparse(substitute(RNA_set))
labelling_csv <- paste("expression", "_for_heatmap_geneset_", set_name, ".csv", sep = "")
labelling_svg <- paste("heatmap", "_for_", set_name, ".svg", sep = "")

#save csv as record
write.csv(RNA_set, file = labelling_csv)

svglite(filename = labelling_svg, width = 6, height = 6, pointsize = 8)
pheatmap(RNA_set,
                   show_rownames = TRUE, show_colnames = TRUE,
                   scale = "row", cluster_cols = FALSE, cluster_rows = clusternon,
                   annotation_row = annotation_file,
                   border_color = NA, fontsize = 8,
                   width = 1.5, height = 6,
                   annotation_colors =  annotation_colors)
dev.off()}

pheatmap_def(RNA_SASP, TRUE, COP_annotation, ann_colors)

pheatmap_def(RNA_CYCLE, TRUE, COP_annotation, ann_colors)

pheatmap_def(RNA_REPAIR, TRUE, COP_annotation, ann_colors)

pheatmap_def(RNA_ACOSTA, TRUE, RNA_ACOSTA_annot, ann_ACOSTA)
```

#WNT/b-catening signalling analysis

```
#TCF/LEF gene check---------------
TCFLEF <- c("TCF7","LEF1","TCF7L1","TCF7L2")
RNA_GOI <- RNA_tidy %>% filter(SYMBOL %in% TCFLEF)
ggplot(data = RNA_GOI, aes(x = SYMBOL, colour = treatment, shape = passage)) +
  theme(legend.position = "top")+
  geom_quasirandom(aes(y = counts), size = 2, show.legend = TRUE) +
```

```
  scale_y_continuous(name = "counts")+
  scale_color_manual(values = c("#4971B6","#CC6666"),
                     labels = c("vehicle","WNT3A"))

#WNT family------------
##because many WNT family members show no/very low expression, do not filter by counts here
genelist.WNT <- read_csv("WNT_family.csv")

## Rows: 19 Columns: 2

## -- Column specification --------------------------------------------------------
## Delimiter: ","
## chr (1): symbol
## dbl (1): order

##
## i Use `spec()` to retrieve the full column specification for this data.
## i Specify the column types or set `show_col_types = FALSE` to quiet this message.

genelist.WNT$ordsymbol <- reorder(genelist.WNT$symbol, genelist.WNT$order)
RNA_WNT <- RNA_integer %>% filter(symbol %in% genelist.WNT$symbol) %>% dplyr::select(symbol, 4:15) %>%
  #here we first split the sample IDs as before into the conditions they encode
  pivot_longer(cols = 2:13, names_to = "ID") %>%
  separate(ID, into = c("treatment","passage","donor"), sep = "_") %>%
  #here we first re-unite the parts of the sample IDs encoding passage and treatment
  ##this just copies above renaming scheme, otherwise splitting could be done more efficiently
  mutate(sample = case_when(
  passage == "P1" & treatment == "FC" ~ "P1 vehicle",
  passage == "P1" & treatment == "FW" ~ "P1 WNT3A",
  passage == "P4" & treatment == "FC" ~ "P4 vehicle",
  passage == "P4" & treatment == "FW" ~ "P4 WNT3A")) %>% inner_join(genelist.WNT, by = "symbol") %>% arrange((ord
er))

plot.WNT_family <- ggplot(data = RNA_WNT, aes(y = value+1, x = sample, colour = sample)) +
  theme(legend.position = "top")+
  theme(axis.text.x = element_text(angle = -90, hjust = 0.5, vjust = 1))+
  geom_beeswarm(aes(), groupOnX = TRUE, size = 1, alpha = 0.5) +
  scale_y_continuous(name = "counts+1", trans = "log10")+
  scale_x_discrete(limits  =  c("P1 vehicle","P4 vehicle","P1 WNT3A","P4 WNT3A"))+
  scale_colour_manual(values = c("light blue","dark blue","pink","dark red"))+
  facet_wrap(vars(ordsymbol))

#BCAT GSEA--------------
##import gene sets
genests_selected <- c("GOBP","HALLMARK","KEGG","RANDOM")
geneset.WNT_multi<- read_csv("WNT_signalling_multiple_V1.csv") %>% filter(term %in% genests_selected)

## Rows: 1264 Columns: 2

## -- Column specification --------------------------------------------------------
## Delimiter: ","
## chr (2): term, symbol

##
## i Use `spec()` to retrieve the full column specification for this data.
## i Specify the column types or set `show_col_types = FALSE` to quiet this message.

#Convert data into named vector
## feature 1: numeric vector
ranked.P4vsP1 <- res_tim_anot$log2FoldChange
ranked.FCvsFW_P1 <- res_WNT_P1_anot$log2FoldChange
## feature 2: named vector
names(ranked.P4vsP1) <- as.character(res_tim_anot$SYMBOL)
```

```r
names(ranked.FCvsFW_P1) <- as.character(res_WNT_P1_anot$SYMBOL)
## feature 3: decreasing order
ranked.P4vsP1 <- sort(ranked.P4vsP1, decreasing = TRUE)
ranked.FCvsFW_P1 <- sort(ranked.FCvsFW_P1, decreasing = TRUE)

#GSEA
GSEA_P1vsP4 <- GSEA(ranked.P4vsP1,
                exponent = 1,
                pvalueCutoff = 1,
                pAdjustMethod = "holm",
                TERM2GENE = geneset.WNT_multi,
                TERM2NAME = NA,
                verbose = TRUE,
                seed = FALSE,
                by = "fgsea")

## preparing geneSet collections...

## GSEA analysis...

## leading edge analysis...

## done...

#GSEA
GSEA_FCvsFW_P1 <- GSEA(ranked.FCvsFW_P1,
                   exponent = 1,
                   pvalueCutoff = 1,
                   pAdjustMethod = "holm",
                   TERM2GENE = geneset.WNT_multi,
                   TERM2NAME = NA,
                   verbose = TRUE,
                   seed = FALSE,
                   by = "fgsea")

## preparing geneSet collections...

## GSEA analysis...

## leading edge analysis...

## done...

#plot GSEA
##combined plot
plot.GSEA_FCvsFW_P1 <- gseaplot2(GSEA_FCvsFW_P1,
                            geneSetID = genests_selected,
                            subplots = 1,
                            base_size = 8,
                            pvalue_table = FALSE,
                            ES_geom = "line")
##combined plot
plot.GSEA_P1vsP4 <- gseaplot2(GSEA_P1vsP4,
                            geneSetID = genests_selected,
                            subplots = 1,
                            base_size = 8,
                            pvalue_table = FALSE,
                            ES_geom = "line")

plot.GSEA_FCvsFW_P1_P <- gseaplot2(GSEA_FCvsFW_P1,
                              geneSetID = genests_selected,
                              subplots = 1,
                              base_size = 8,
```

```
                                pvalue_table = TRUE,
                                ES_geom = "line")
##combined plot
plot.GSEA_P1vsP4_P <- gseaplot2(GSEA_P1vsP4,
                             geneSetID = genests_selected,
                             subplots = 1,
                             base_size = 8,
                             pvalue_table = TRUE,
                             ES_geom = "line")

#because display is slow in Rstudio, plot with Cairo instead as raster files
Cairo(width = 7, height = 4, units = "in", dpi = 300, file = "GSEA_WNTsignalling_P1vsP4.png", type = "png")
plot.GSEA_P1vsP4_P
```

```
dev.off()

Cairo(width = 7, height = 4, units = "in", dpi = 300, file = "GSEA_WNTsignalling_FWvsFC_P1.png", type = "png")
plot.GSEA_FCvsFW_P1_P

dev.off()

#assemble patchwork
patch.WNT_bcat <- {plot.WNT_family | plot.GSEA_FCvsFW_P1 / plot.GSEA_P1vsP4}
svglite(filename = "WNT_bcat.svg", width = 8, height = 5)
patch.WNT_bcat
dev.off()
```

#senescence and quiescence genes

```
#senescence markers
genetab.senescence <- read_csv("geneset_senescence_up_down_multi.csv")
genetab.quiescence <- read_csv("geneset_senescence_vs_quiescence_allq.csv")

#reshape  data to suit lollipop plotting format
RNA_fold <- RNA_tidy %>% pivot_wider(names_from = c("passage","treatment"), values_from = counts) %>%
  mutate(          fold_WNT_P1 = (P1_FC+1)/(P1_FW+1),
                   fold_WNT_P4 = (P4_FC+1)/(P4_FW+1),
                   fold_tim_FC = (P4_FC+1)/(P1_FC+1)) %>% group_by(SYMBOL) %>%
  mutate(across(c(fold_WNT_P1,fold_WNT_P4,fold_tim_FC), mean, .names = "mean_{.col}"))

popplot_GOI <- function(dataset, GOI_list, GOI){
labelling_svg <- paste("popplot", "_for_", "senescence_", GOI, ".svg", sep = "")
#generate list with genes of interest
RNA_list <- dataset %>% filter(SYMBOL %in% GOI_list$symbol) %>%
  inner_join(.,GOI_list, by = c("SYMBOL" = "symbol")) %>% filter(source == GOI)%>% arrange(category)

#plot for WNT at P4
plot.pop_WNT <- ggplot(data = RNA_list, aes(x = SYMBOL)) +
  theme(legend.position = "top")+
  geom_point(aes(y = fold_WNT_P4, colour = category), alpha = 0.5, size = 2, stroke = 0, show.legend = FALSE)+
  geom_segment(aes(x = 0, xend = Inf, y = 1, yend = 1),
             size = 0.5, linetype = "dotted", colour = "black", show.legend = FALSE)+
  geom_point(mapping = aes(y = mean_fold_WNT_P4, colour = category),
             shape = 18, size = 2, alpha = 1, show.legend = FALSE)+
  geom_segment(aes(x = SYMBOL, xend = SYMBOL, y = mean_fold_WNT_P4, yend = 1, colour = category),
  show.legend = FALSE)+
  scale_y_continuous(name = "fold Vehicle/WNT (P4)", trans = "log2", labels = label_number())+
  scale_x_discrete(name = "")+
  theme(axis.line = element_line(size = 0.5))+
  theme(axis.text.x = element_text(angle = -45))+
  theme(legend.text = element_blank())
```

```
#plot for P1 -> P4 in vehicle
plot.pop_tim <- ggplot(data = RNA_list, aes(x = SYMBOL)) +
  theme(legend.position = "top")+
  geom_point(aes(y = fold_tim_FC, colour = category), alpha = 0.5, size = 2, stroke = 0)+
  geom_segment(aes(x = 0, xend = Inf, y = 1, yend = 1),
             size = 0.5, linetype = "dotted", colour = "black", show.legend = FALSE)+
  geom_point(mapping = aes(y = mean_fold_tim_FC, colour = category),
             shape = 18, size = 2, alpha = 1, show.legend = FALSE)+
  geom_segment(aes(x = SYMBOL, xend = SYMBOL, y = mean_fold_tim_FC, yend = 1, colour = category),
  show.legend = FALSE)+
  scale_y_continuous(name = "fold P4/P1 (vehicle)", trans = "log2", labels = label_number())+
  theme(axis.title.x = element_blank(), axis.text.x = element_blank())+
  theme(axis.line = element_line(size = 0.5))+
  scale_x_discrete()

#assemble into overview image
patch <- plot.pop_tim / plot.pop_WNT
return(patch)}


svglite(filename = "popplot_senescence_common.svg" ,width = 1.5, height = 4)
popplot_GOI(RNA_fold,genetab.senescence,"common")
dev.off()

svglite(filename = "popplot_senescence_Hernandez-Segura.svg" ,width = 3, height = 4)
popplot_GOI(RNA_fold,genetab.senescence,"Hernandez-Segura")
dev.off()

svglite(filename = "popplot_senescence_Casella.svg" ,width = 3, height = 4)
popplot_GOI(RNA_fold,genetab.senescence,"Casella")
dev.off()

svglite(filename = "popplot_senescence_quiescence.svg" ,width = 2, height = 4)
popplot_GOI(RNA_fold,genetab.quiescence,"quiescence")
dev.off()
```
