# Supplementary material for: WNT/beta-catenin signalling interrupts a senescence-induction cascade in human mesenchymal stem cells that restricts their expansion
Source: Cell Mol Life Sci. 2022 Jan 20;79(2):82. doi: 10.1007/s00018-021-04035-x (PMC8770385; doi:10.1007/s00018-021-04035-x)
Supplement: Supplementary file 5 — Supplementary file4 (DOCX 2821 KB) [file 18_2021_4035_MOESM5_ESM.docx]

Supplementary Data

| **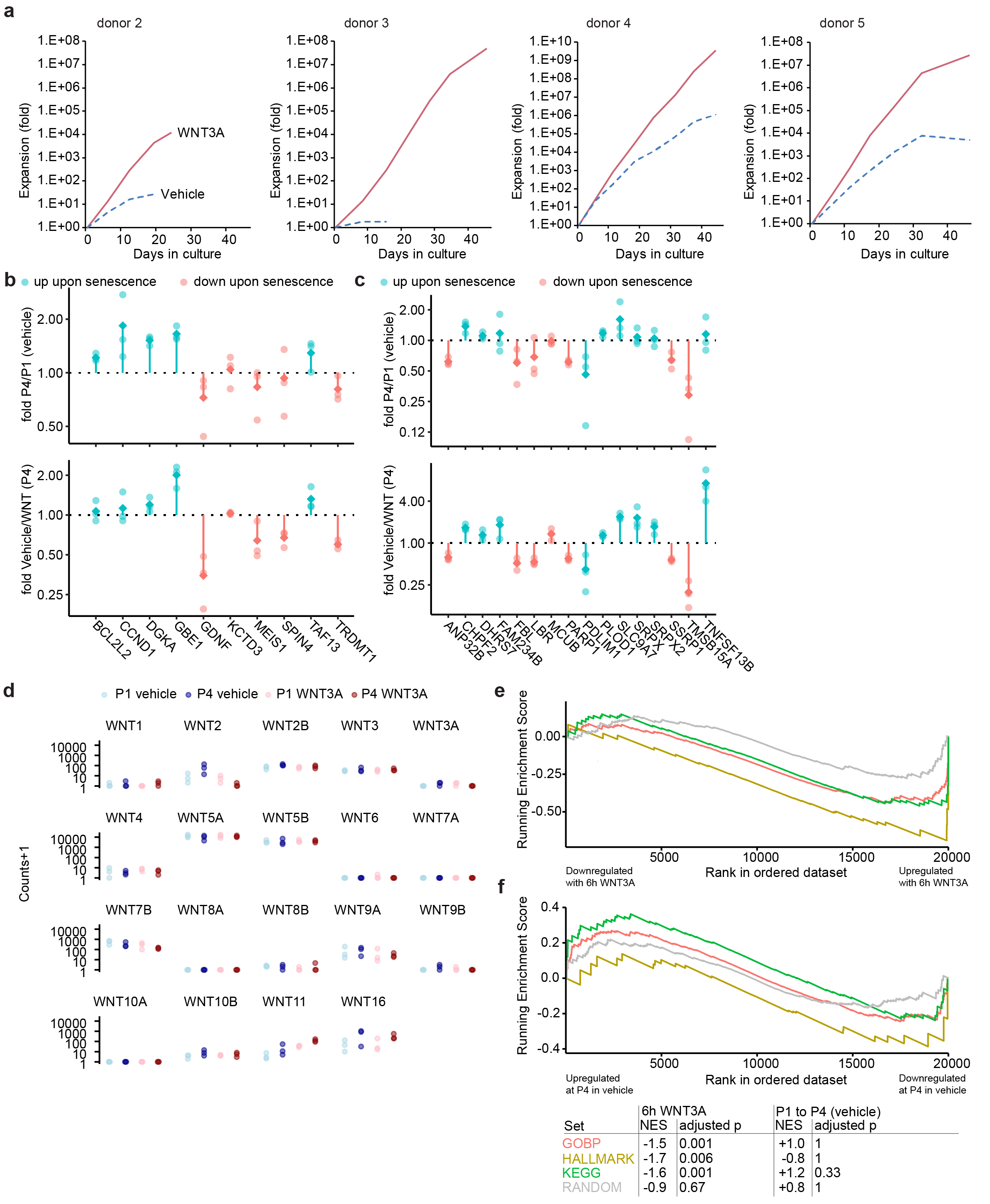** |
| --- |
| **Supplementary Figure 1 \| WNT3A maintains cell cycle drivers and DNA repair** (**a**) Expansion for MSCs from different donors cultured with WNT3A or vehicle. **(b, c)** Expression of genes up- and down-regulated upon senescence based on (**b**) Hernandez-Segura et al. 2017 [49] and (**c**) Casella et al. 2019 [48] in vehicle expanded MSCs at passage four as a as a fold of their expression at passage one in vehicle (top) or at passage four in WNT3A (bottom). *n = 3 donors.* (**d**) Expression of WNT ligand family members (as RNAseq counts+1) plotted for MSCs at passage 1 treated with vehicle or WNT3A for 6 hours or at passage 4 expanded with vehicle or WNT3A from passage 1 on. (**e, f**) Gene set enrichment analysis for three WNT/beta-catenin signalling pathway gene sets and a set of random genes with normalized enrichment score (NES) and multiple testing adjusted (Holms-Bonferroni) p-values given for (**e**) MSCs at passage 1 treated for 6 hours with WNT3A treatment compared to vehicle as positive control for WNT/beta-catenin signalling activity or (**f**) MSCs in vehicle at P1 compared to at P4. WNT/beta-catenin signalling pathway gene sets shown are: GOBP (Gene Ontology biological process: canonical WNT signalling), KEGG (Kyoto Encyclopedia of Genes and Genomes: WNT signalling pathway) and HALLMARK (Molecular Signatures Database Hallmark Genesets: WNT/beta-catenin signalling). |
| 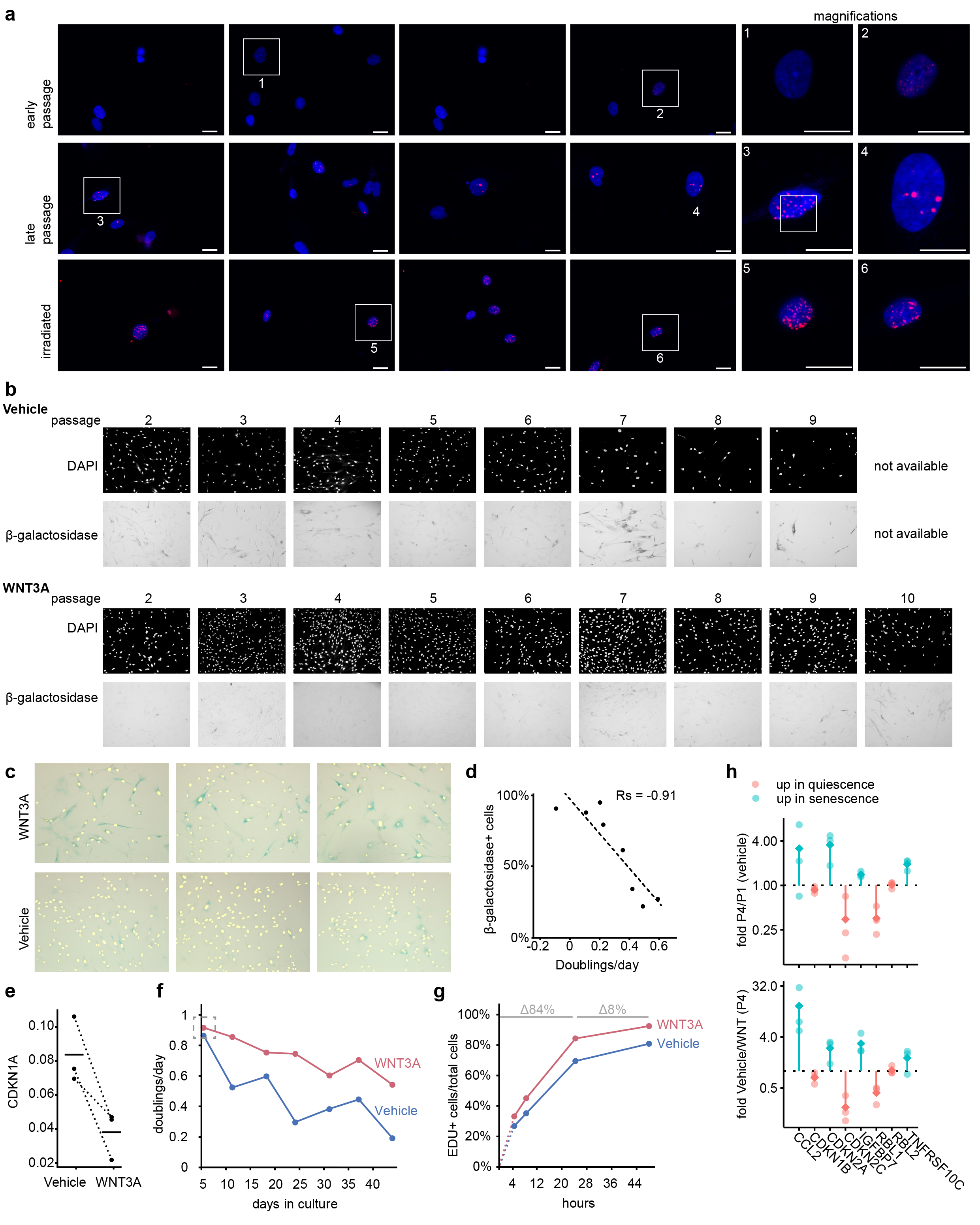 |
| **Supplementary Figure 2 \| WNT3A reduces the frequency of entry into senescence** Representative micrographs of MSCs after expansion for two to three passages (early) or seven to nine passages (late) or after irradiation stained for γH2AX (red) and DAPI (blue). *Scale bars = 20 µm. Magnified images on the right-hand size are representative for nuclei with no foci [1] and small foci (early passage, [2]) or large foci (late passage [3,4] and irradiated [5,6]).* (**b**) Micrographs for senescence-associated β-galactosidase and DAPI staining of MSCs expanded with WNT3A or vehicle over ten passages. Images representative of 3 wells imaged per passage. *Note: expansion of vehicle cultured MSCs was too low at passage 9 to allow further passaging.* (**c**) Micrographs for β-galactosidase activity and DAPI (*inverted* *to yellow*). staining of MSCs expanded with WNT3A or vehicle as in (b). Images from passage 6, (**d**) Scatter plots with linear trendline comparing percentage of β-galactosidase positive cells to the number of doublings a day in one donor over multiple passages. *Rs _=_ Spearman’s rank correlation coefficient.* (**e**) mRNA expression of *CDKN1A* relative to expression of housekeeping genes (*HPRT, GAPDH)* in MSCs expanded for three passages in the indicated conditions. *n= 3 donors (paired by lines). (****f****)* Doublings per day plotted against time in culture with either vehicle or WNT3A, with the initial rate (grey box) 0.87 population doublings/day for vehicle and 0.92 doublings/day with WNT3A *(n = 1 donor). (****g****)* Percentage of MSCs incorporating EDU at the indicated time points in culture with WNT3A or vehicle with the change in percentage points within the first and the second 24-hour period given *(n = 1 donor, dotted line extrapolates to 0). (***h***)* Expression of genes up-regulated in senescence and genes up-regulated in quiescence (but not in both) based on He and Sharpless 2017 [56] and Wiley et al. 2017 [127] in vehicle expanded MSCs at passage four as a as a fold of their expression at passage one in vehicle (top) or at passage four in WNT3A (bottom). *n = 3 donors.* |
| *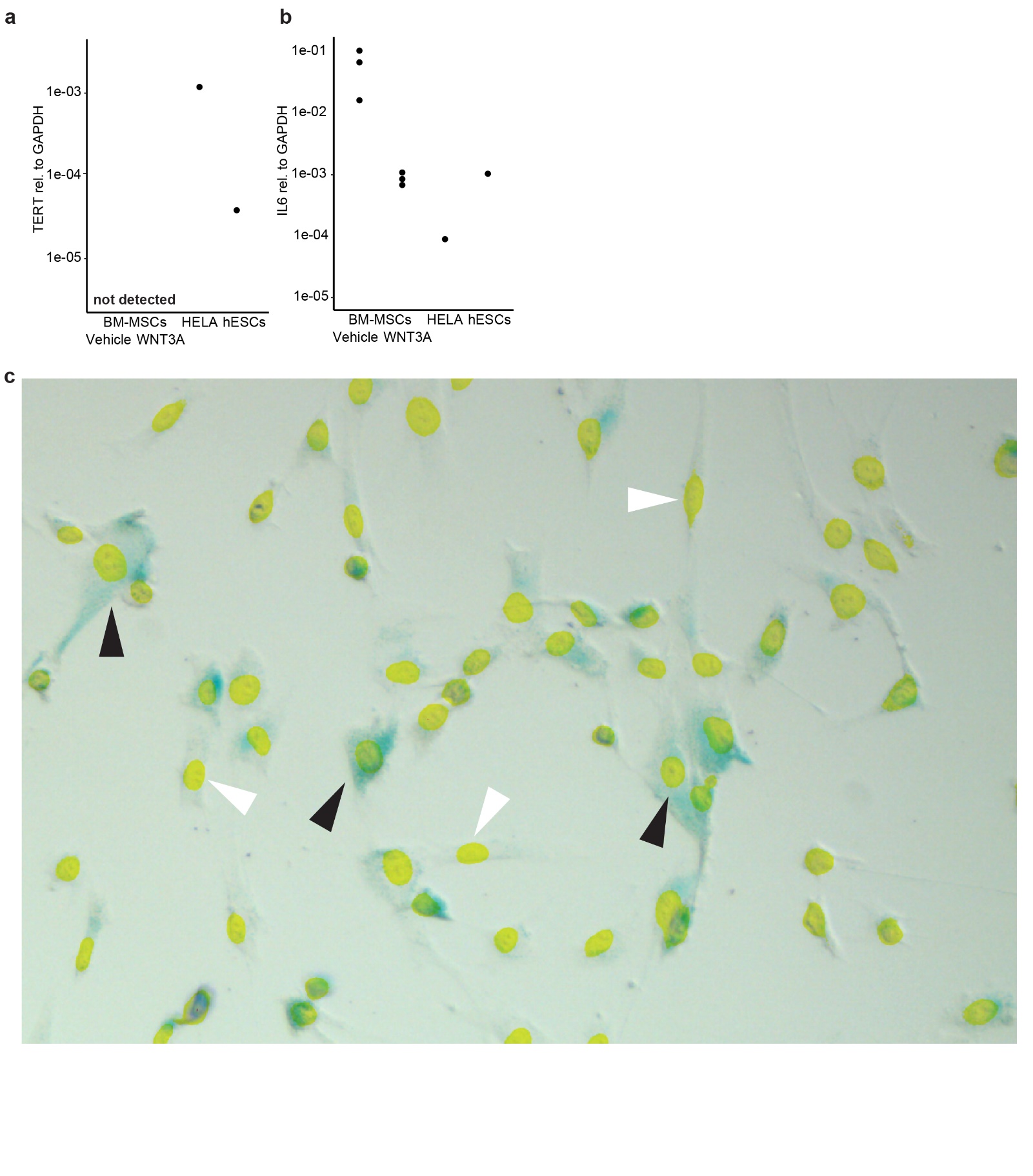* |
| **Supplementary Figure 3 \| WNT represses senescence in a cell non-autonomous manner.** (**a**) Expression of TERT relative to GAPDH as determined by QPCR for MSCs expanded in vehicle or WNT3A or for HELA cells and human embryonic stem cells. (**b**) Expression of IL6 relative to GAPDH and HPRT for the same samples run in parallel as control for (**b**). *n = 3 donors (MSCs), n = 1 (HELA, hESC).* (**c**) Micrograph of representative vehicle expanded clone stained for β-galactosidase activity and DAPI (*inverted* *to yellow*). White arrow heads = β-galactosidase negative, black arrow heads = β-galactosidase positive. |
| **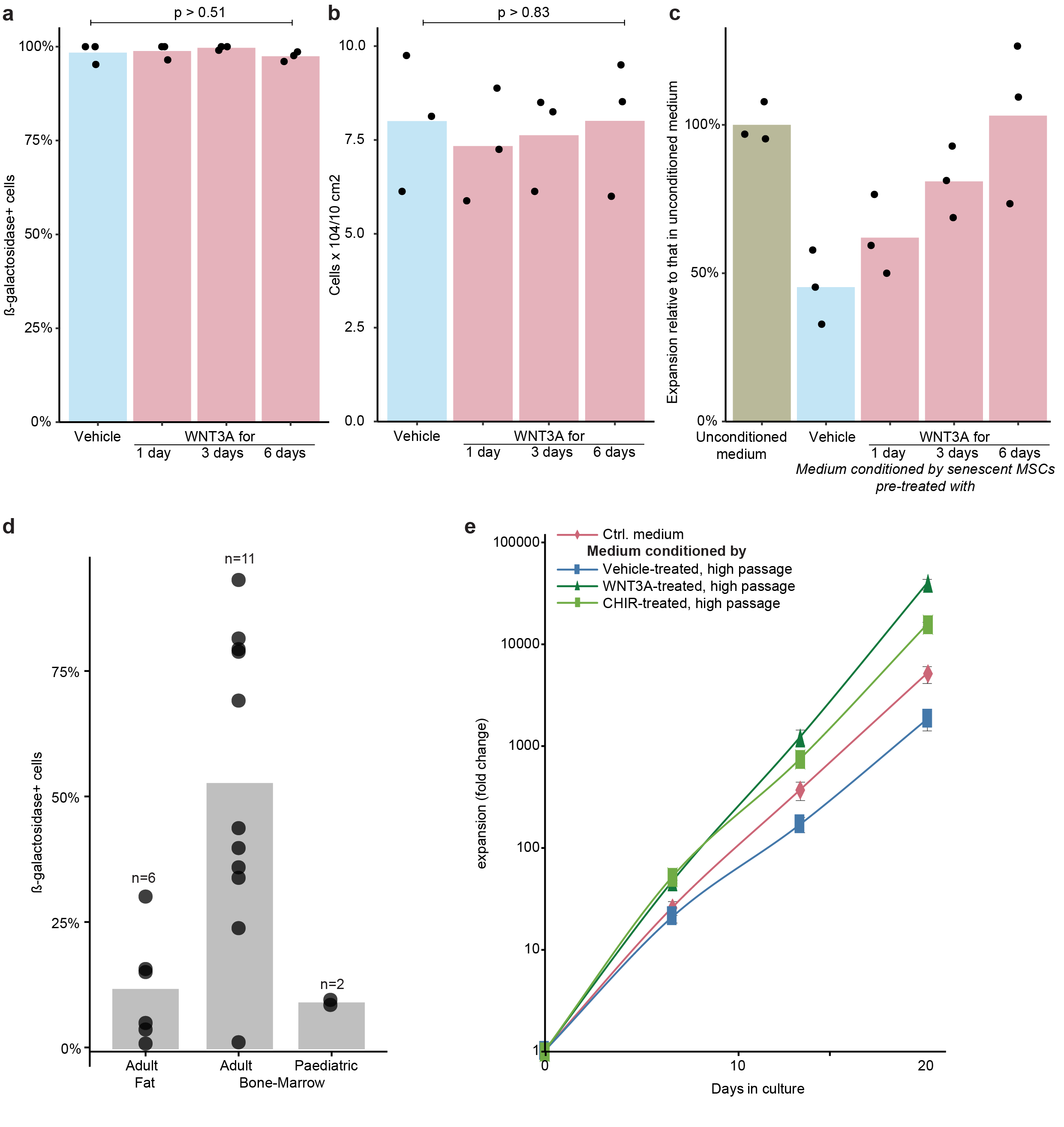** |
| **Supplementary Figure 4 \| WNT/β-catenin signaling represses paracrine senescence**  (**a**) Percentage of β-galactosidase positive cells and (**b**) cell numbers and for MSCs two weeks after irradiation with 80 Gray [conditioning cells from (c)] after treatment for one, three or six days with WNT3A or six days with vehicle. (a, b) *n = 3 donors, p-values from Wilcoxon-Mann-Whitney* *test.* (**c**) Expansion of passage one MSCs in control medium or medium conditioned by MSCs irradiated with 80 Gray and treated one, three or six days with WNT3A or six days with vehicle. (**d**) Percentage of β-galactosidase positive cells in adult fat-derived MSCs, adult bone marrow-derived MSCs and paediatric bone marrow-derived MSCs after 2-3 weeks in culture. (**e**) Expansion of passage one MSCs in control medium or medium conditioned by vehicle, CHIR99021 or WNT3A treated late passage MSCs. *n = 3 donors, error bars = SD.* |
| **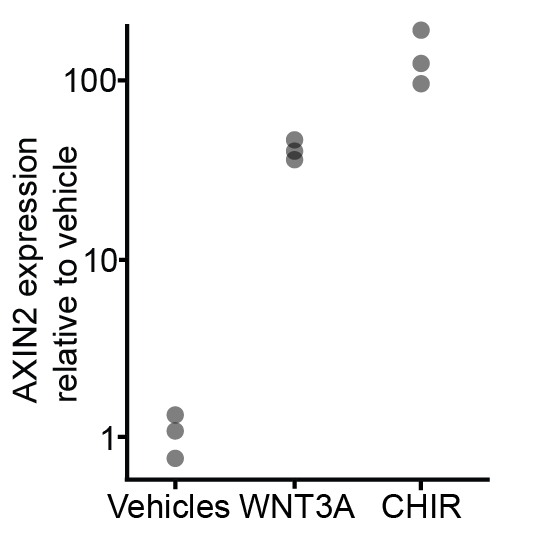** |
| **Supplementary Figure 5 \| WNT/β-catenin pathway activation represses factors mediating paracrine senescence**  Expression of AXIN2 determined by QPCR for MSCs cultured for 24 hours with vehicles, WNT3A or CHIR99021. |
